# Whole-genome sequencing reveals novel tandem-duplication hotspots and a prognostic mutational signature in gastric cancer

**DOI:** 10.1038/s41467-019-09644-6

**Published:** 2019-05-02

**Authors:** Rui Xing, Yong Zhou, Jun Yu, Yingyan Yu, Yongzhan Nie, Wen Luo, Chao Yang, Teng Xiong, William K. K. Wu, Zhongwu Li, Yang Bing, Shuye Lin, Yaping Zhang, Yingqi Hu, Lin Li, Lijuan Han, Chen Yang, Shaogang Huang, Suiping Huang, Rui Zhou, Jing Li, Kaichun Wu, Daiming Fan, Guangbo Tang, Jianhua Dou, Zhenggang Zhu, Jiafu Ji, Xiaodong Fang, Youyong Lu

**Affiliations:** 1 Laboratory of Molecular Oncology, Key Laboratory of Carcinogenesis and Translational Research (Ministry of Education), Peking University Cancer Hospital & Institute, 100142 Beijing, China; 2 Institute of Digestive Disease and Department of Medicine and Therapeutics, State Key Laboratory of Digestive Disease, Li Ka Shing Institute of Health Sciences, CUHK Shenzhen Research Institute, Shenzhen, 518000 China; 3 Department of Surgery, Rui-jin Hospital, Shanghai Jiaotong University School of Medicine, Shanghai, 200025 China; 4 State Key Laboratory of Cancer Biology, Xijing Hospital of Digestive Diseases, Fourth Military Medical University, Shanxi, 710032 China; 5 Shanghai Clinical Center for Endocrine and Metabolic Diseases, Shanghai Key Laboratory for Endocrine Tumours, Rui-Jin Hospital, Shanghai Jiao-Tong University School of Medicine, 200025 Shanghai, China; 6 The Second Affiliated Hospital of Guangzhou University of Chinese Medicine, 510120 Guangzhou, China; 7 College of Life Science and Technology, Huazhong Agricultural University, 430070 Wuhan, China; 8 Department of Medical Oncology, Beijing Hospital, 100730 Beijing, China

**Keywords:** Cancer, Computational biology and bioinformatics, Biomarkers, Health care, Oncology

## Abstract

Genome-wide analysis of genomic signatures might reveal novel mechanisms for gastric cancer (GC) tumorigenesis. Here, we analysis structural variations (SVs) and mutational signatures via whole-genome sequencing of 168 GCs. Our data demonstrates diverse models of complex SVs operative in GC, which lead to high-level amplification of oncogenes. We find varying proportion of tandem-duplications (TDs) among individuals and identify 24 TD hotspots involving well-established cancer genes such as *CCND1, ERBB2* and *MYC*. Specifically, we nominate a novel hotspot involving the super-enhancer of *ZFP36L2* presents in approximately 10% GCs from different cohorts, the oncogenic role of which is further confirmed by experimental data. In addition, our data reveal a mutational signature, specifically occurring in noncoding region, significantly enriched in tumors with *cadherin 1* mutations, and associated with poor prognoses. Collectively, our data suggest that TDs might serve as an important mechanism for cancer gene activation and provide a novel signature for stratification.

## Abstract (web summary)

Structural variations in gastric cancer impact progression. Here, the authors perform whole-genome sequencing on 168 gastric cancer patients and identified tandem-duplications of super-enhancer ZFP36L2 in 10% of gastric cancer, and mutational signatures in tumors with cadherin 1 mutations that associated with poor prognoses.

## Introduction

Gastric cancer (GC) is the second most common cancer and the third leading cause of cancer death in China. The advent of next-generation sequencing has rapidly expanded our knowledge of the genetic basis of this disease, and many studies have provided useful and cross-validated information concerning the genes and classifications potentially representing therapeutic targets^1–3^. These studies provided novel insights into GC from the perspective of gene mutations and the underlying mutational mechanisms. However, most of these studies are based on exome sequencing or focused on coding mutations, despite the availability of whole-genome sequencing (WGS). Therefore, structural variations and genome-wide mutational signatures derived from WGS analysis still need to be elucidated.

Structural variations (SVs) that often lead to the disruption of tumor suppressors and the amplification of oncogenes play an important role in tumorigenesis and malignant phenotypes. SV-formation mechanisms, especially those related to complex SVs, account for a large proportion of high-level amplifications and have been described in many cancers, but are relatively less studied for GC. These complex SVs and SV-affected genes require investigation in the context of GC. Additionally, tandem duplication (TD), leading to only one extra copy of some reference-sequence regions as compared with multiple copies derived from complex SVs, is often omitted in cancer genomics studies. Menghi et al. described a chromotype termed as a TD phenotype (TDP) characterized by frequent and distributed TDs and significantly enriched in triple-negative breast, serous ovarian, and endometrial carcinomas^4^. Nik-Zainal et al. further characterized the genomic landscape of breast cancer using 560 whole-genome sequences and identified three distinct SV signatures associated with different sizes of TDs or deletions, subsequently identifying 33 hotspots associated with large (>100 kb) TDs enriched in breast cancer germline-susceptibility loci and super-enhancer regulatory elements^5^. Similarly, Zhang et al. analyzed the whole-genome sequences of 12 tumor types and demonstrated that TD-induced amplification of super-enhancer could activate driver genes^6^. These findings suggest that, different from high-level gene amplifications that directly increase oncogene copy number, TDs upregulate oncogene expression by increasing the copy number of super-enhancer regions. Therefore, it is important to discover novel genes driven by TDs in GC.

Here, we present WGS data from 168 GC patients derived in association with the International Cancer Genome Consortium project to describe the SVs landscape of GC in Chinese population. We describe diverse models of SV and affected genes in GC, of which we unveil a TD hotspot involving the coding sequence and super-enhancer region of zinc finger protein 36 ring-finger protein-like 2 (*ZFP36L2*).

## Results

### Characterization of GC samples and analytic approach

We collected GC primary tumor tissue from 168 patients that had not received prior chemotherapy or radiotherapy in China (Supplementary Data 1). Of these, GC tumors could be divided into intestinal and diffuse types based on Lauren classification (91 and 77 tumors, respectively). Compared with the intestinal type, diffuse-type tumors were associated with poorer survival (*P* = 0.008; Log-rank test; Supplementary Fig. 1A). Additionally, 143 (85.12%) tumors have clearly tumor location within the stomach (19.05% in cardia, 20.24% in fundus/body, and 45.83% in angular notch/antrum/pylorus; Supplementary Fig. 1B). Tumors of different regions showed distinct prognoses, with the survival of proximal tumors (cardia and fundus/body) significantly poorer than distal tumors (angular notch/antrum/pylorus) (*P* = 0.011; Log-rank test; Supplementary Fig. 1C). Moreover, the clinical stage and MSI status were also observed as prognostic factors in this cohort (Supplementary Fig. 1D, E). We also inferred TCGA-based EBV, MSI and CIN subtypes based on our 168 GCs (Online Method), and these four types of GCs tend to be different in overall survival (Supplementary Fig. 1F). These results were consistent with previous reports^7, 8^ and suggested that our GC cohort was suitable for further study.

### Distribution of driver genes across 168 GCs

Overall, somatic mutation burden in the 168 GC samples varied greatly (Supplementary Data 1), from 0.7 to 195.9 somatic single-nucleotide variant (SNVs) per Mb (Supplementary Fig. 2A), and could be divided into hypermutated and regular-mutated GCs by unsupervised clustering (Online Method). In particular, only *TP53* (54.76%) gene was significantly mutated with a false discovery rate of <0.1 evaluated by the algorithm *MutSigCV*^9^. Except for *TP53*, 12 previously reported genes, including *APC* (8.93%), *MUC6* (8.33%), *ARID1A* (8.33%), *GLI3* (7.74%), *PIK3CA* (7.74%), *RNF43* (7.74%), *CDH1* (7.74%), *KRAS* (6.55%), *CTNNA2* (4.17%), *RHOA* (2.38%), *ZIC4* (1.79%) and *PTEN* (1.19%), were found in less than 10% GCs in this study (Supplementary Fig. 2A).

Copy number changes of 168 GCs were profiled to identify significant peaks that might contain potential driver genes (Supplementary Fig. 2B). We have observed a high frequency of arm-level changes, including gains of 7p, 8q, 13q, 20p, 20q and losses of 4p, 4q, 17p, 21q, and 22q (Supplementary Fig. 2C), most of which were consistent with previous results^1^. Simultaneously, we identified 80 recurrent amplified and 124 deleted regions (Supplementary Fig. 2D), including some previously reported significantly mutated genes *EGFR*, *MYC*, *CCND1*, *ERBB2*, *CCNE1*, and *CDKN2A* (Supplementary Fig. 2D). Particularly, we identified two novel focal amplification peaks containing *ZFP36L2* gene and *MYB* gene, respectively.

### Somatic structural variations across 168 GCs

**Fig. 1:**
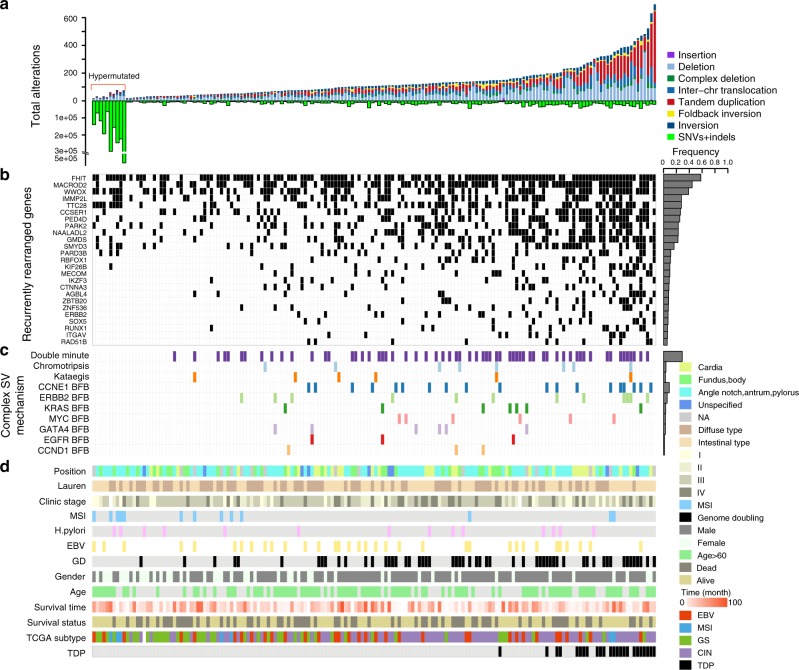
Summary of SVs in GC. **a** The total number of structural changes (above the 0 axis) and point mutations or indels (below the 0 axis) for 168 GC specimens resident in hypermutated and regular-mutated GCs. **b** Genes from at least ten samples that underwent rearrangement. **c** Presence of localized complex SVs. **d** Clinical characteristics of 168 GC patients. SV structural variation, GC gastric cancer

A total of 23,264 somatic SVs were detected from the 168 GC genomes at an average of 138 SVs per tumor (range: 19–696; Fig. 1a and Supplementary Data 2), including 8430 (36.2%) deletions, 6363 (27.4%) TDs, 3642 (15.6%) inter-chromosomal translocations, 2696 (11.6%) inversions, 1131 (4.9%) complex deletions, 914 (3.9%) fold-back inversions, and 88 (0.4%) insertions. To verify the accuracy of SVs, 60 predicted SVs were randomly chosen and PCR and Sanger sequencing were done. As a result, 95% (57/60) of the selected SVs were validated (Supplementary Fig. 3). The frequency of somatic SVs is variable in each sample, and not related to SNVs, as hypermutated GCs had a lower number of SVs compared to regular-mutated GCs. Consistent with the accumulating evidence that genome doubling (GD) is associated with chromosomal instability, we found that a higher prevalence of SVs was observed in GCs with GD events (*P* < 0.001; Wilcoxon rank sum test, two side; Fig. 1d). We also found an association between SV and gender, as 36/42 patients with top quartile of SVs were male (*P* < 0.001; Wilcoxon rank sum test, two side; Fig. 1d). However, the underlying reason for this result remains unclear.

Across 168 GC samples, the breakpoints of 15,038 rearrangements were found to directly disrupt gene sequences. We observed many recurrently rearranged genes, and there were 25 genes with SVs occurring in at least ten tumors (Fig. 1b). The most frequently affected genes were in fragile sites, such as *fragile histidine triad protein* (57.9%), *MACRO domain containing 2* (44.4%), and *WW domain containing oxidoreductase* (38.8%). Some previously reported oncogenes, such as *SET and MYND domain containing 3* (16.3%), *Erb-B2 receptor tyrosine kinase 2* (*ERBB2*) (7.0%), and tumor suppressors, such as *RNA-binding protein, Fox-1 homolog 1* (10.7%), *catenin A3* (8.4%), *SRY-Box 5* (7.0%), *runt-related transcription factor 1* (6.0%), and *RAD51 paralog B* (*RAD51B*) (5.6%), were also detected in our cohort. We then searched for functional fusion genes, revealing that a total of 811 in-frame fusion genes and 1148 out-of-frame fusion genes were the result of somatic rearrangements but without evidence of recurrent, potentially driver events in GC (Supplementary Data 3).

### Complex structural variations patterns in GC

Highly complex focal events were observed in the GCs in this study, including double minute-like chromosomes (62/168), chromothripsis with a one-off genomic catastrophe (8/168), high-level amplifications with a breakage-fusion-bridge (BFB) pattern, and kataegis with locally rearranged SVs (6/168). Amplifications of the oncogenes *fibroblast growth factor receptor 2* (*FGFR2*) (4/168) and *CTNNB1* (2/168) were derived from double minutes in our cohort (Supplementary Fig. 4), and eight samples suffered from chromothripsis involving chr1, chr2, chr12, and chr17 (Supplementary Fig. 5). Moreover, high-level amplifications (≥5 copies) of numerous oncogenes, such as *cyclin E1 (CCNE1)* (19/168), *ERBB2* (12/168), *endothelial growth factor receptor* (*EGFR*) (3/168), *MYC* (6/168), *KRAS* (7/168), *GATA-binding protein 4* (6/168), and *CCND1* (3/168), were derived from BFB cycles (Fig. 1c; Supplementary Fig. 6A). The 19q21 region harboring *CCNE1* was the most prominent signal from high-level amplification and colocalized with fold-back inversions, indicating that most of the *CCNE1* amplification was a result of BFB cycles (Supplementary Fig. 7). Furthermore, kataegis analysis detected localization of a hypermutation region with multiple rearrangements in six tumors (Supplementary Figs. 6B, 8). Interestingly, kataegis events occurred at least twice in each of six GCs. These data revealed complex focal SVs existed in the GC specimens.

### Tandem-duplication phenotypes in GC

**Fig. 2:**
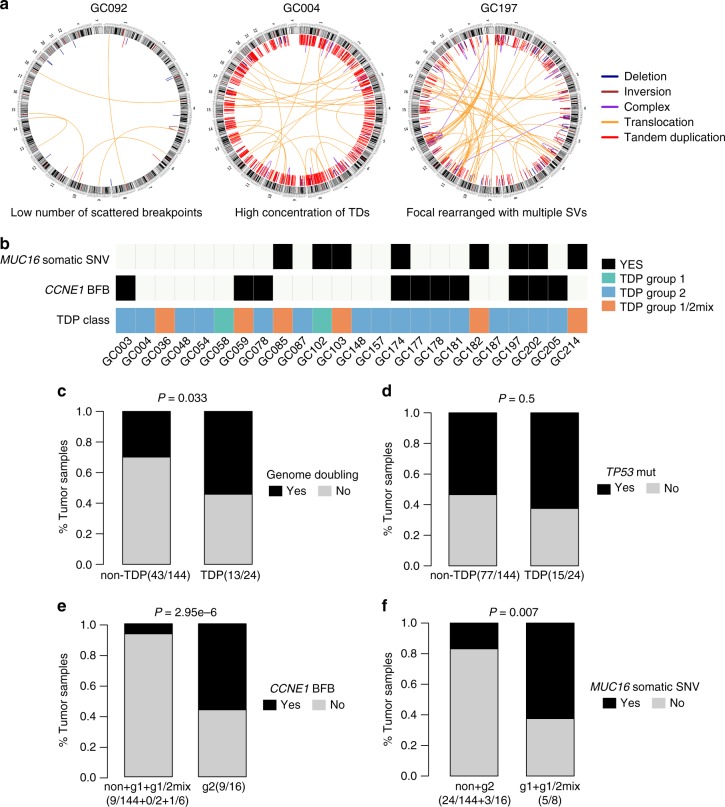
Classification of TDP in GC. **a** Circos plots describing somatic rearrangements, with chromosomal ideograms located on the outermost right side and lines representing rearrangements. **b** Colored squares indicate *MUC16* somatic mutation status, *CCNE1* BFB status and TDP status of 24 TDP GCs. The below bar plots showed comparison of TDP in GCs with or without GD (c) and TP53 mutation (**d**). The bottom bar plots exhibited comparison of TDP subgroups and oncogenic drivers: **e** TDP group 2 and *CCNE1* BFB status; **f** TDP group 1 and 1/2mix and *MUC16* somatic mutation status. *P* values were derived from Fisher’s exact tests. TDP tandem duplication phenotype, GC gastric cancer, BFB breakage-fusion-bridge

The distribution of SVs within each tumor was complex. Some GCs had a low number of scattered breakpoints, whereas others had a high concentration of particular SV types, such as deletions or TDs. Many of these SVs represented highly complex rearrangements with multiple classes of rearrangement (Fig. 2a), indicating distinctive underlying mutational processes. Notably, several GCs showed a high proportion of TDs, which were evenly distributed throughout the entire genome and similar to the tandem duplicator phenotypes (TDPs) identified in a previous study^4^. Fourteen per cent (24/168) of the tumors were classified as harboring TDPs (TD-proportion range: 20–74%, mean: 42.4%, Supplementary Fig. 9A; Online Method). TDs associated with these TDPs were more likely to occur within gene bodies than in intergenic regions (Supplementary Fig. 9B). Moreover, we observed that the size distribution among the TDs was strikingly different and followed a trimodal pattern (Supplementary Fig. 9C). TDs were generally classified into three categories: <100 kb, 100 kb to 1 Mb, or >1 Mb. We then compared the breakpoint junctions between TDP and non-TDP types and three span-size categories, finding the borderline significance according to microhomology patterns (Supplementary Fig. 9D). These differences indicated distinct mechanisms underlying TD formation. However, we did not identify a significant association between TDP and clinical metrics, such as prognosis and TCGA molecular subtypes (Supplementary Fig. 9E; Fig. 1d). In addition, 58% (14/24) of the TDP GCs were found to be CIN subtypes, which may be attributed to the fact that CIN GCs generate more SVs. This result was further supported by the fact that TDP is significantly associated with genome doubling (*P* = 0.033, Fisher’s exact test; Fig. 2c).

Using the method described by Menghi et al.^10^, we further classified TDP-type GCs into three groups based on modal patterns of span size in each GC: TDP group 1 with a TD peak ~11 Kb (2/24), TDP group 2 with a TD peak ~231 Kb (16/24) and group 1/2 mix with both TD peaks around 11 and 231 Kb (6/24; Fig. 2b). Statistical association between *TP53* mutational status and TDP revealed by Menghi was not observed in our study (Fig. 2d). We also found no association between TDP groups and BRCA status, which may be due to the low mutation rate of *BRCA1/2* and the lack of methylation data. By contrast, there is a strong correlation between TDP group 2 and *CCNE1* BFB events in our data (*P* < 0.0001, Fisher’s exact test, Fig. 2e). Moreover, *CCNE1* was not perturbed by group 2 TDs with a peak of ~231 Kb; it was amplified by BFB or coefficient of BFB and large size TDs with >1 Mb (Supplementary Figure 7), supporting that *CCNE1* amplifications driven by BFB events may be a cause of TDP group2. Furthermore, we compared individual gene somatic mutations and TDP groups, and discovered that *MUC16* was strongly linked to the TDP group 1 and 1/2 mix profile (*P* = 0.007, Fisher’s exact test; Fig. 2f). Taken together, these results suggested these oncogenic drivers may induce a specific pattern of structural rearrangements (TDP), which can serve as a biomarker in clinical decision.

### Tandem-duplication hotspots

TDs always give rise to amplification in genes within the rearranged region. Frequent TDs in a particular region of different individual tumors indicate that there might be driver genes that associated with tumorigenesis curated in this hotspot region. Here, we used an approach described by Glodzik et al. to identify TD hotspots associated with the three TD categories in GC^11^. Interestingly, 2767 short TDs (<100 kb) occurred at two hotspots, whereas 2727 modest TDs (100 kb–1 Mb) formed at 17 hotspots, and 869 long TDs (>1 Mb) were located at five hotspots (Supplementary Data 4). Most of these hotspots overlapped with GISTIC focal-amplification peaks, such as those associated with *ERBB2* (33/168), *MYC* (18/168), *Kruppel-like factor 5* (*KLF5*) (10/168), *cluster of differentiation 44* (10/168), *CCND1* (9/168), and *MYB* (6q12, 7/168), described in TCGA and suggesting that TD hotspots effectively increased the copy number of numerous well-described driver amplicons that might confer a selective advantage. The broadly distributed TDs in the TDP GCs targeted two or more cancer genes, indicating that genome-wide TD formation might contribute to carcinogenesis (Supplementary Fig. 9F). Specifically, we observed that two hotspots occurred in the vicinity of the noncoding regions of *ZFP36L2* and *MYC*, which usually harbor enhancers that can be bound by transcription factors to activate oncogenes.

### Oncogenic function of ZFP36L2 in GC

*ZFP36L2* is a member of an RNA-binding protein family and exhibits opposing roles in different tumor types, playing an oncogenic role in pancreatic cancer^12^, but displaying tumor-suppressor activity in esophageal squamous-cell carcinoma (ESCC)^13^; however, the biological relevance of *ZFP36L2* in GC remains unknown.

**Fig. 3:**
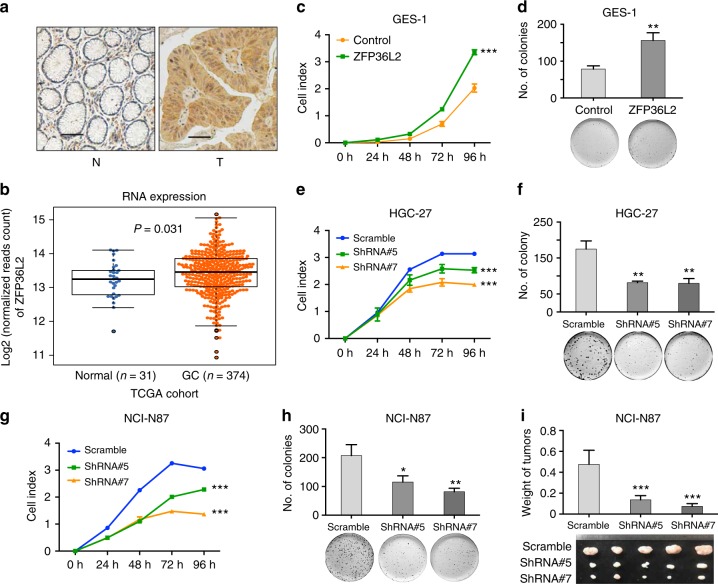
Highly expressed *ZFP36L2* induced by amplification promotes cell growth. **a** Immunohistochemistry detection of *ZFP36L2* expression in GC samples with amplification. Scale bars 25 μm. **b** Comparison of *ZFP36L2* mRNA expression between matched nonmalignant tissues and tumor samples in the TCGA GC cohort. Center line represents the median of mRNA expression and *P* value was derived from Wilcoxon rank-sum test. **c**, **d** Overexpression of *ZFP36L2* significantly promoted cell growth in GES-1 cells, the normal gastric epithelial cell line, as monitored by **c** RTCA and **d** colony formation assay. *P* values were derived from t tests. **P ≤ 0.01; ***P ≤ 0.001. Error bars represent ± s.d. of three experiments. **e**, **f** Knock-down of *ZFP36L2* significantly inhibited cell growth in HGC-27 cells, a GC cell line, as monitored by **e** RTCA and **f** colony formation assay. *P* values were derived from t tests. **P ≤ 0.01; ***P ≤ 0.001. Error bars represent ± s.d. of three experiments. **g**, **h** Knock-down of *ZFP36L2* significantly inhibited cell growth in NCI-N87 cells, a GC cell line, as monitored by **g** RTCA and **h** colony formation assay. *P* values were derived from t tests. *P ≤ 0.05; **P ≤ 0.01; ***P ≤ 0.001. Error bars represent ± s.d. of three experiments. **i** The photograph of tumors obtained from nude mice injected with NCI-N87 cell transfected with shRNAs against ZFP36L2 or control. *P* values were derived from *t* tests. ***P ≤ 0.001. Error bars represent ± s.d. of five nude mice GC gastric cancer

A total of 18 GC-amplification sites contained *ZFP36L2* or neighboring noncoding regions, of which amplifications in eight GCs encompassed both the gene body and neighboring noncoding regions. As shown in Fig. 3a and Supplementary Fig. 10, 18 GCs with ZFP36L2 rearrangements had significantly higher protein expressions compared to that in paired normal tissues (*P* < 0.001; Chip-Squared Test). Additionally, we observed that *ZFP36L2* was more highly expressed in GC specimens than in noncancerous tissues in a TCGA GC cohort (Fig. 3b). To characterize the effects of *ZFP36L2* on cellular function, we stably overexpressed *ZFP36L2* in the normal gastric epithelial cell line, GES-1 cells, expressing low levels of *ZFP36L2* (Supplementary Fig. 11A), xCELLigence RTCA DPlus System showed that stably overexpressed *ZFP36L2* significantly promoted cell growth (Fig. 3c). A colony formation assay showed a significant increase in the colony formation of cells overexpressing *ZFP36L2* (Fig. 3d). We also stably knocked-down *ZFP36L2* in a GC cell line, HGC-27 cells (Supplementary Fig. 11B), and xCELLigence RTCA DPlus System showed that knocked-down *ZFP36L2* significantly inhibited cell growth (Fig. 3e). A colony formation assay showed a significant decrease in the colony formation of cells knocked-down *ZFP36L2* (Fig. 3f). Similar results were replicated in NCI-N87 cells, another GC cell line (Supplementary Fig. 11C and Fig. 3g, h). Further, xenograft experiment showed that knock-down of *ZFP36L2* inhibited the growth of NCI-N87 cells in vivo (Fig. 3i). These results suggested a potential oncogenic role of *ZFP36L2* in GC.

### The ZFP36L2 super-enhancer harbors a TD hotspot

**Fig. 4:**
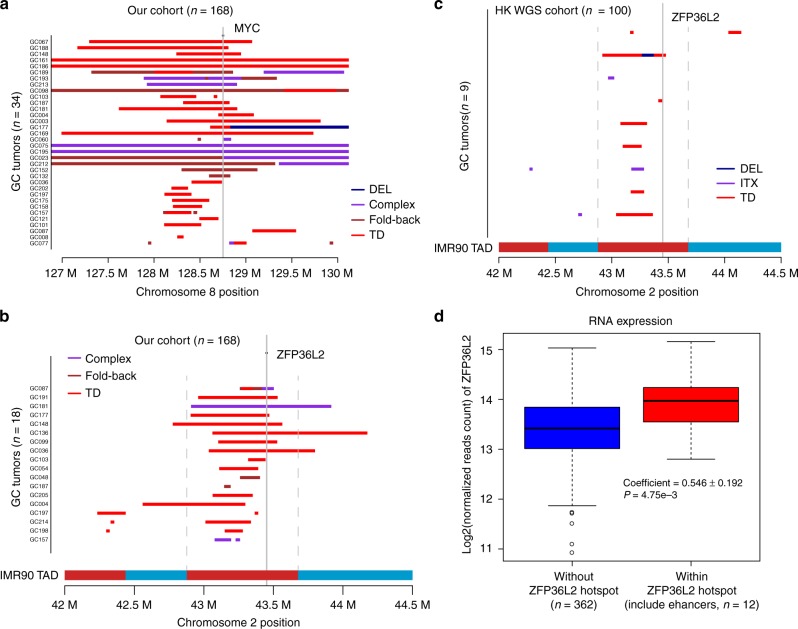
Amplification of *ZPF36L2* and *MYC* super-enhancers. **a** Rearrangements localized in the *MYC*-coding and -enhancer regions. **b** Rearrangements localized in the *ZPF36L2-*coding and neighboring-noncoding regions. Chromatin interaction, measuring by Hi-C in the cell line IMR90, is presented in the vicinity of *ZFP36L2* locus. The topologically associated domains (TAD) are shown as adjacent red and blue bars. **c** Validation of TD-induced amplification of *ZFP36L2* super-enhancers in an HK WGS cohort (*n* = 100). **d** Comparison of *ZFP36L2* expression between samples with or without focal amplification in the TCGA GC cohort. Center line represents the median of mRNA expression and *P* value was derived from Wilcoxon rank-sum test. TD tandem-duplication

**Fig. 5:**
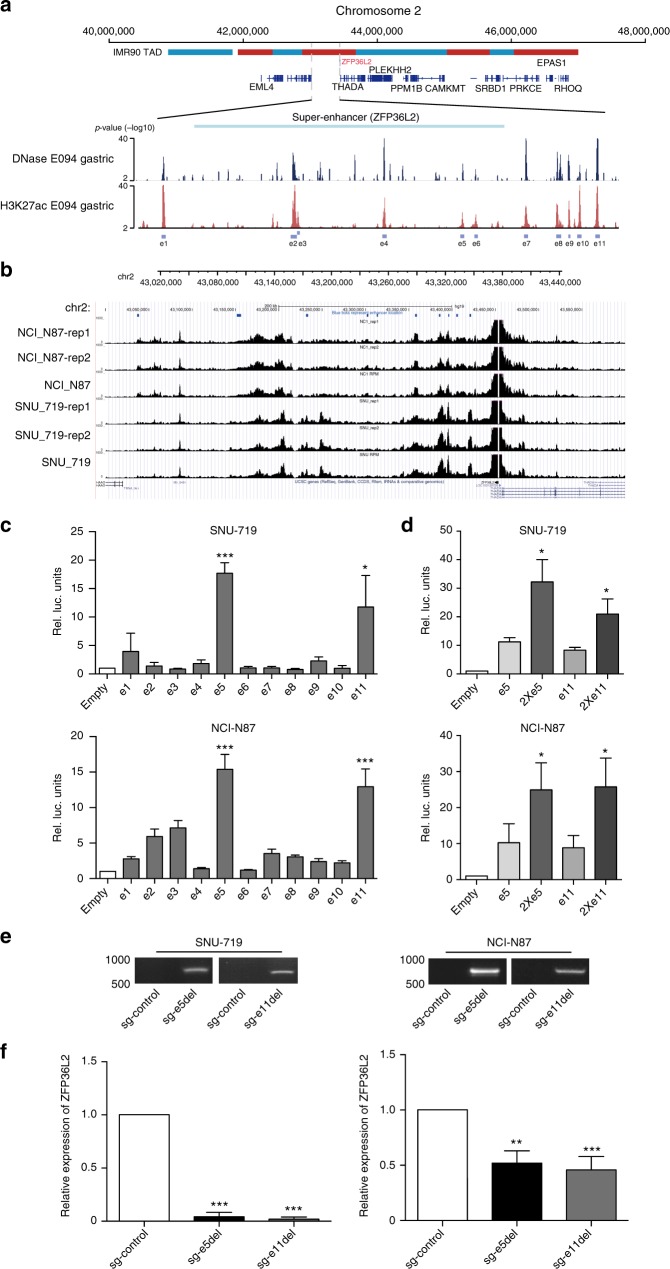
*ZFP36L2* expression is predominantly driven by the e5 and e11 constituent enhancers. **a** H3K27ac and DNase-hypersensitivity profiles in E094 cells to identify constituent enhancers e1 through e11 within the super-enhancer region. **b** Capture-4C experiment assays showed that the super-enhancer region (chr2:43036808-43422165, from e1 to e11) physically interacts with the *ZFP36L2* promoter. All of the Capture-C experiments for each cell line were performed with two biological replicates. **c** Luciferase-reporter assays (*n* = 3) measuring the activity of e1 through e11 in (upper) SNU-719 cells and (lower) NCI-N87 cells. The pGL3 plasmid without the enhancer region (empty) was used as a negative control. Along the *Y*-axis, the luciferase signal was first normalized to the Renilla luciferase signal and then normalized to the signal from the empty pGL3 plasmid. *P* values were derived from *t* tests. *P ≤ 0.05; ***P ≤ 0.001. **d** Enhancer activity of duplicated e5 (2 × e5) and e11 (2 × e11) enhancers measured by luciferase-reporter assays (*n* = 3) in SNU-719 cells and NCI-N87 cells. *P* values were derived from *t* tests. *P ≤ 0.05. **e** Gel pictures of PCR amplification of genomic DNA using primers outside the e5 and e11 enhancer region in (left) SNU-719 and (right) NCI-N87 cells with CRISPR/Cas9-mediated deletion of the e5 and e11 enhancer. sg-Control: empty plasmid; sg-e5del and sg-e11del: pairs of sgRNAs recognizing boundaries of the e5 and e11 enhancer region. **f** The expression level of ZFP36L2 as measured by quantitative PCR in (left) SNU-719 and (right) NCI-N87 cells with CRISPR/Cas9-mediated deletion of the e5 and e11 enhancer. *P* values were derived from *t* tests. **P ≤ 0.01; ***P ≤ 0.001. Error bars represent ±s.d. of three experiments

TDs of *MYC* super-enhancers were previously identified in human epithelial cancers and associated with overexpression of the *MYC* oncogene^6^. Our data showed that 13 GCs contributed to the *MYC* hotspot, with single or nested rearrangements occurring in the *MYC* super-enhancer (Fig. 4a). Additionally, we identified *ZFP36L2* amplification in a downstream region in 18 GC tissues from our cohort (Fig. 4b). Intriguingly, in 9% of the HK WGS cohort, we also identified amplification of a downstream region of *ZFP36L2* (Fig. 4c), of which 78% were derived from TDs. As amplifications involving the downstream region of *ZFP36L2* had similar SV patterns as those of the *MYC* super-enhancer in our cohort, we anticipated that the focal-amplified downstream regions of *ZFP36L2* might harbor super-enhancers targeting the *ZFP36L2* gene. Since these amplifications overlapped in a region nearly 500 kb downstream of *ZFP36L2*, we downloaded DNase-seq data and H3K27ac chromatin immunoprecipitation (ChIP)-seq data from the corresponding gastric tissue and identified 11 enhancers, denoted e1 through e11 here, in the 500 kb regions downstream of *ZFP36L2* (Fig. 5a; Supplementary Data 5). Interestingly, in a previous study, Hnisz et al. reported evidence of a super-enhancer containing enhancers e8 through e11 in gastric tissue according to H3K27ac ChIP-seq (Supplementary Data 5)^14^, which further indicated the presence of a curated super-enhancer in the downstream region of *ZFP36L2*. As the overlapped region spanned beyond e8 through e11 to as far as 500-kb downstream, to better understand the probable enhancers associated with *ZFP36L2*, we broadly defined regions e1 through e11 as a super-enhancer in GC. Additionally, we observed focal amplification of *ZFP36L2* super-enhancers in TCGA GC and EAC cohorts (Supplementary Fig. 12)^15^. *ZFP36L2-*super-enhancer amplification showed trends for anatomical-location associations, as seven of ten tumors (70%; *P* = 0.178; Fisher’s exact test; our cohort) and five of nine tumors (55.6%; *P* = 0.385; Fisher’s exact test HK WGS cohort) presented in the distal region of the stomach.

Enhancers regulate gene expression through physical interaction with gene promoters and these interactions are contained within the topologically associating domain (TAD), which are highly conserved across tissue types^16^. Utilizing publicly available Hi-C data from IMR90 lung fibroblast cells that measures physical interaction between chromatin regions and defines TADs in the genome^17^, we found that the amplified super-enhancer was within the same TAD (chr2:42,880,000−43,680,000) as the promoter region and gene body of *ZFP36L2* (Fig. 5a). Furthermore, the majority of rearrangements or amplifications involving *ZFP36L2* from both our data and HK-WGS data was located within the same TAD (Fig. 4, Supplementary Fig. 12).

We also performed Capture-4C experiment assays with the promoter region of *ZFP36L2* (chr2: 43,453,458−43,454,797) as an anchoring point in SNU-719 and NCI-N87 cells and found that most locations of the enhancers show similar peaks as that in H3K27ac data, suggesting that these enhancers have physical interactions with promoter of *ZFP36L2* (Fig. 5b).

### Super-enhancer amplification drives ZFP36L2 overexpression

To determine whether super-enhancer amplification drives oncogene expression, we first analyzed TCGA transcriptome data, finding that GCs with focal amplification of *ZPF36L2* super-enhancers are associated with increases in *ZFP36L2* expression (Fig. 4d). We then cloned super-enhancers into a PGL3-promoter plasmid based on H3K27ac peaks and performed luciferase-reporter assays in SNU-719 and NCI-N87 cells. Our results showed that the e5 and e11 enhancers had the strongest activity (Fig. 5c). Duplication of the e5 and e11 enhancers in the luciferase-reporter construct resulted in >2-fold higher luciferase expression relative to a single copy of e5 and e11, demonstrating that an increase in copy number of the enhancer region might upregulate target-gene expression (Fig. 5d).

To investigate the functional role of the e5 and e11 enhancer region in ZFP36L2 expression, we used the CRISPR/Cas9 system to specifically delete the e5 and e11 enhancers, respectively. Single guide (sg) RNAs were used to target Cas9 to the boundaries of the e5 and e11 enhancers. Deletion of e5 and e11 was detected by PCR (Fig. 5e and Supplementary Fig. 13). Deletion of the e5 and e11 enhancer region resulted in a reduction in *ZFP36L2* expression (Fig. 5f). These data supported that the oncogenic role of *ZFP36L2* is primarily driven by tandem duplication of its super-enhancer.

### Signature 18* in GC

**Fig. 6:**
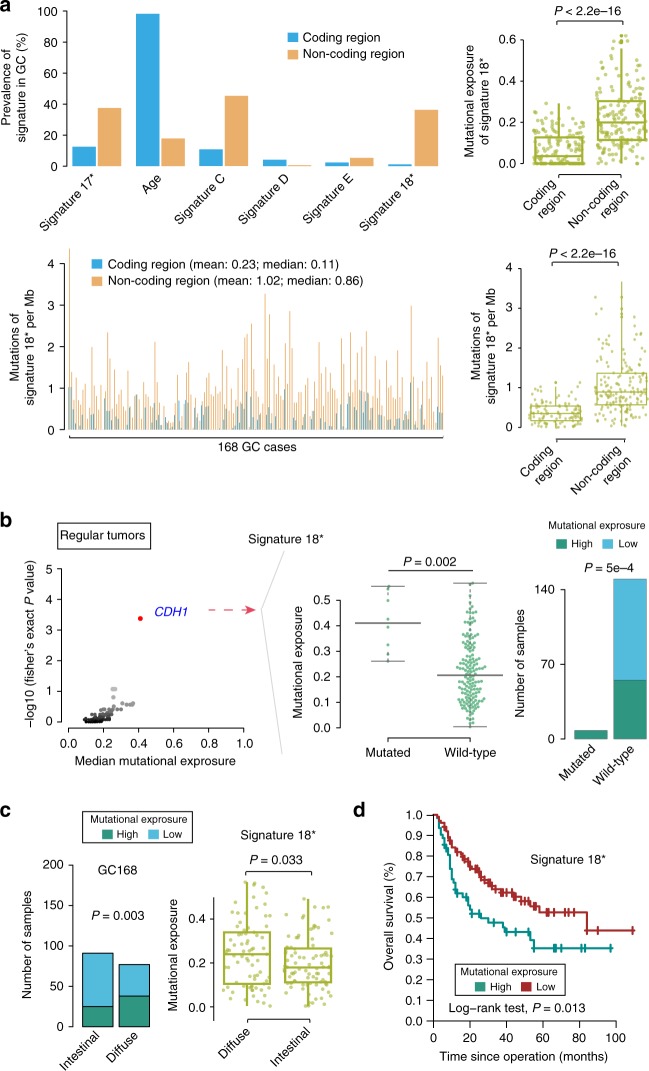
Summary of the presence of mutational signature 18* in 168 GC specimens. **a** Prevalence of each mutational signature and mutation of signature 18* per Mb across GCs between noncoding and coding regions. In the left panel, the *X*-axis shows the signatures or each sample, and the *Y*-axis shows the proportion of samples or mutation of signature 18* per Mb in the coding or noncoding region. Mutational exposure or mutation of signature 18* per Mb of signature 18* in the coding or noncoding region was revealed (right). One dot represents one sample. *P* values were derived from *t* tests. **b** (left) Mutational-exposure analysis revealed an association between somatic *CDH1* mutations (regular tumors referred to those which are without somatic hypermutation) and mutational signature 18*. High mutational-signature contribution was defined as GC with mutational-signature contributions >25%. Genes mutated in >4% of samples were chosen from our cohort. Genes with a false discovery rate (*q*) <0.1 are highlighted in red. *CDH1* was the only gene showing a significant difference. The *P* value for evaluating mutational-exposure between *CDH1* status was derived from *t* tests. Center line of boxplot represents the median of mutational exposure of signature 18*. **b** (right) The contribution of signature 18* was compared in wild-type versus mutated tumors. *P* values were derived from Fisher tests. **c** Relationship between signature 18* and diffuse-type GC in our cohort. One dot represents one sample. *P* values were derived from Fisher tests and *t* tests. **d** Kaplan−Meier survival curves show the survival outcomes of signature 18* in GC. High mutational-signature contribution represents mutational-signature contributions ≥25%. GC gastric cancer

We also analyzed the genome-wide mutational spectrum of GC. The mutational spectrum in a trinucleotide context showed different mutational patterns between coding and noncoding SNVs. C > T transition at CpG sites increased, whereas both C > A and T > G mutations decreased in coding regions as compared with noncoding regions. Similar patterns in coding regions were also confirmed in a TCGA-exome cohort (Supplementary Fig. 14A). These findings suggested distinct mutational processes in coding and noncoding regions. We further compared the prevalence of six signatures, identified from whole-genome mutations (Supplementary Fig. 14B), between coding and noncoding regions, revealing that the prevalence was obviously different for signature 17*, aging signature, and signature 18* (Fig. 6a). The aging signature was more likely driven from the coding region, whereas signature 17*, 18*, and the ABOPEC signature were more prevalent in the noncoding region. Specially, signature 18* almost exclusively occurred in the noncoding region (*P* < 0.001; Student’s *t* test, two side; Fig. 6a). The mutation rate of signature 18* shows a significant elevation in noncoding compared to that in coding region (*P* < 0.001; Student’s *t* test, two side). This phenomenon is also evident in the HK-WGS cohort (Supplementary Fig. 15A). These findings illustrated the variable extent of variation in the contribution of mutational processes between coding and noncoding regions.

Interestingly, GC with *CDH1* mutations had high mutational exposure to signature 18* that was also associated with diffuse-type GC in both our data and the HK-WGS cohort (Fig. 6b, c; Supplementary Fig. 15B). Kaplan−Meier survival analysis showed that signature 18* predicted a worse prognosis (Fig. 6d), and multivariate analysis revealed that the prognostic significance of signature 18* was independent of age, sex and TNM staging (Supplementary Fig. 15C). In diffuse type, GCs with signature 18* tend to have worse outcome compared to counterparts (Supplementary Fig. 15D). Moreover, only 15.4% (2/13) and 25% (3/12) of *CDH1* mutations in our cohort and HK cohort harbored a C > A substitution, which is the main feature of signature 18* (Supplementary Fig. 15E). In fact, only 15.9% (10/63) of GCs with signature 18* have *CDH1* mutations. In addition, signature 18* was prevalent in the whole-genome region of EAC^18^ and coding region of neuroblastoma^19^, whereas *CDH1* mutation rarely occurred in these two types of cancer. These data supported the hypothesis that *CDH1* mutation is a cause rather than a consequence of signature 18*. Further studies are needed to elucidate the biological relevance of *CDH1* mutations in GC signature 18*.

## Discussion

In the present study, we analyzed SVs by performing WGS on specimens from 168 GC patients along with complete clinical information. We described the SV landscape of GCs and demonstrated complex SVs involving well-established driver genes such as *CCNE1*, *EGFR*, *MYC* and the novel drivers *ZFP36L2*.

SVs, especially those occurring in super-enhancer regions, also have the potential to deregulate several oncogenes and are clearly associated with malignant phenotypes^20, 21^. Previously, Zhang et al. reported that copy number gains in noncoding regions harboring super-enhancers near *MYC* are associated with its overexpression in lung cancer^6^. They also reported focal amplification of super-enhancer-activated *KLF5* expression in squamous-cell carcinomas. In the present study, we also revealed a TD hotspot in the super-enhancer region of *MYC* and *ZFP36L2*. Both our and TCGA data showed that TD-induced focal amplification of *ZFP36L2* super-enhancers were associated with an increase in *ZFP36L2* expression. Notably, the SV and *ZFP36L2* expression and function differ between adenocarcinoma and squamous-cell carcinoma. The focal amplification of *ZFP36L2* super-enhancers was observed in our cohort, as well as the HK, the TCGA GC, and the EAC cohorts, but not in ESCC, the *ZFP36L2* super-enhancer was hypermethylated^13^. Consistent with altered super-enhancer, *ZFP36L2* expression was higher in adenocarcinoma, but lower in squamous-cell carcinoma^13^. Our functional study showed that highly expressed *ZFP36L2* promoted the growth of GC cells, with this phenomenon also observed in pancreatic ductal adenocarcinoma cells by Yonemori et al., whereas Lin et al. showed that low expression of *ZFP36L2* promoted the growth of OSCC cells^12, 13^. These data suggested that *ZFP36L2* might exert opposite functions in different cancer cells.

Several molecular-characterization studies focusing on coding regions have been conducted in a GC context; however, it is commonly known that variants in intergenic, promoters and intronic regions can strongly influence phenotypic outcomes. Through analysis of the mutational spectrum, we showed different mutational patterns between coding and noncoding SNVs. Specifically, signature 18* occurred exclusively in the noncoding region, leading to an increase in C > A mutations and providing an underlying rationale for the failure to detect signature 18* in previous GC exome-sequencing data^8, 19^. Subsequently, survival analysis showed that signature 18* was associated with poor prognosis. These data revealed that the noncoding region is capable of classifying tumors and predicting prognosis in GC.

## Methods

### Sample selection

Clinical sample collection was conducted in accordance with the Helsinki Declaration and was approved by the Institutional Ethical Standards Committee at Peking University Cancer Hospital/Institute, with informed consent provided by the patients. GC samples were collected from the Beijing Cancer hospital, Xijing hospital, Shanghai Ruijin hospital, and CUHK Shenzhen Research Institute. DNA was extracted from 168 GC tissues, as well as the adjacent matched normal tissues. Samples were subjected to quality review, and selected cases needed to exhibit an estimated carcinoma content of >60% for inclusion in the study.

### Whole-genome sequencing (WGS)

DNA from both tumors and adjacent matched normal tissues were sequenced using an Illumina HiSeq sequencer (Illumina, San Diego, CA, USA), with 100 or 150 bp paired-end reads. Raw reads were subjected to SOAPnuke (v1.5.0; https://github.com/BGI-flexlab/SOAPnuke) processing to remove sequencing adapters and low-quality reads. High-quality reads were aligned to the National Center for Biotechnology Information human reference genome (hg19) using the Burrows−Wheeler Aligner (v0.5.9; http://bio-bwa.sourceforge.net/). Duplicate marking was performed using Picard (v1.54; http://broadinstitute.github.io/picard/), and to improve alignment accuracy, the Genome Analysis Toolkit (v1.0.6076; GATK IndelRealigner; https://software.broadinstitute.org/gatk/)^22^ was used within the cohort. Sequencing depths for tumors and adjacent matched normal samples were 35.56× and 35.57× on average, respectively, and the median fraction of whole-genome bases was 99.69% for tumors and adjacent matched normal samples. The percentage of the genome covered at 20× was 92.14% for tumors and 92.81% for controls (Supplementary Data 6).

### Structural variation detection and validation

We used Meerkat (http://compbio.med.harvard.edu/Meerkat/)^23^ to predict somatic SVs and their breakpoints using the suggested parameters. This method used soft-clipped and split reads to find candidate breakpoints and refined precise breakpoints by local alignments. We predicted mutational mechanisms for these SVs based on homology and sequencing features. The final somatic SVs were generated by filtering out germline events and other artifacts. Meerkat also generated fusion genes based on these SV breakpoints. We then used SvABA (https://github.com/walaj/svaba; unpublished), a genome-wide local-assembly method, to re-examined the SV data generated by Meerkat. These two methods have an overlapping frequency of >70%, and we discarded SVs that were termed as germline SVs by SvABA.

Sixty somatic SVs were randomly chosen from one sample GC177, and further PCR and Sanger sequencing were performed to validate their accuracy. The priers for each SV are given in Supplementary Data 7.

### TDP analysis

We used a method described by Menghi et al. to calculate a TDP score for each GC^4^. For each tumor sample, we counted the total number of TDs and compared the observed (Obs_*i*_) and expected (Exp_*i*_) numbers of TDs for each chromosome, *i*: ${\mathrm{TDPscore}} = - \frac{{\mathop {\sum }\nolimits_i \left| {{\mathrm{{Obs}}}_i - {\mathrm{{Exp}}}_i} \right|}}{{{\mathrm{TD}}}}$. We set the threshold for distinguishing between non-TDP and TDP tumors to −0.6 based on the TDP-score distribution. Three conditions were used to filter false positive results. Tumors meeting the three criteria were classified as TDP tumors: (1) TDP score ≥ −0.6; (2) TD proportion ≥ 20%; (3) TD number ≥ 50 (Supplementary Fig. 9A).

For each one of the 24 TDP tumors, we computed the span size density distribution of all the detected TDs. Using the normalmixEM function of the mixtools R package, we identified the major peak of the distribution plus any additional peaks whose density measured at least 25% of the distribution mode. We then subgrouped TDP tumors based on the criteria used by Menghi et al.^10^. Tumors featuring a TD span size modal distribution were designated as TDP group 1 or TDP group 2 based on the presence of a single TD span size distribution peak at 1.64−51 Kb or 51−622 Kb, respectively. Similarly, tumors featuring a TD span size bimodal distribution were designated as mix group, such as TDP group 1/2mix (two peaks at 1.64−51 Kb and 51−622 Kb).

To discover the association between somatic SNV and TDP, we used only potentially damaging somatic variants and comprised nonsense, frame-shift, splice site and missense mutations. Candidate genes associated with specific TDP states were considered those whose mutation rate was at least 10% and were specifically associated with TDP subgroup. The significance of the associations was determined via Fisher’s exact test.

### Enrichment of super-enhancer elements

The DNase-seq and H3K27ac ChIP-seq data were downloaded from the National Institutes of Health Roadmap Epigenomics Mapping Consortium (http://egg2.wustl.edu/roadmap), and we extracted *ZFP36L2* super-enhancers active in gastric tissues.

### Somatic mutation calling

The potential somatic SNVs and somatic small insertions or deletions (INDELs) were separately called by MutTect (http://archive.broadinstitute.org/cancer/cga/mutect)^24^ and SvABA (https://github.com/walaj/svaba)^25^ using default parameters based on paired-alignment files (tumor and normal data). These SNVs were filtered supported reads ≥ 4 (≤2) and coverage ≥ 14 (≥10) in tumors and (normal tissue), respectively. Somatic mutations were annotated by Oncotator (https://portals.broadinstitute.org/oncotator/)^26^. For mutation-rate calculation, the number of mutations (consisting of SNVs and small indels) were compared against the total number of bases that sufficiently covered the entire genome. We used MutSigCV (http://software.broadinstitute.org/cancer/software/genepattern/modules/docs/MutSig CV)^9^ to identify significantly mutated genes. Hypermutations were determined using the following method: samples with mutation rates <1 per Mb in coding regions were removed, resulting in *n* (≤168) cases. We then defined a coordinate with points ${\mathrm{Z}}_i\left( {x_i,y_i} \right),i \in \left[ {1, \cdots ,n} \right]$, where *x*_*i*_ refers to the genome-wide mutation rate, and *y*_*i*_ refers to the mutational rate of the coding region. We could then calculate the distance between tumor *i* and tumor *j* as $d_{ij} = \sqrt {(x_i - x_j)^2 + (y_i - y_j)^2}$, with tumor *j* median distance $d_j = {\mathrm{{median}}}(d_{ij})$. We also computed the center of all tumors as $d_{\mathrm{c}} = {\mathrm{{median}}}(d_{ij})$. We calculated the local density, indicating the ratio of cases closer than *d*_c_, using the formula $\rho _j = \frac{{\mathop {\sum }\nolimits_i \chi (d_{ij} - d_{\mathrm{c}})}}{n}$, where $\chi \left( {\it{\epsilon }} \right) = 1$ if ${\it{\epsilon }} < 0$; otherwise $\chi \left( {\it{\epsilon }} \right) = 0$. A tumor was considered hypermutated if it had a low local density and a long median distance.

### Mutational signatures extraction

We applied nonnegative matrix factorization and model-selection approaches^27^ to delineate mutational processes underlying genome-wide SNVs and identified five mutational signatures. In the cohort, one sample was considered as a strong possibility associated with one mutational signature if the proportion of the contribution assigned to its signature was more than 25%. The mutational exposure referred to the sample contribution of a signature. Estimating the contributions of mutational signatures in each sample, coding region, and/or noncoding region was performed using the R package (MutationalPatterns; http://bioconductor.org/packages/release/bioc/html/MutationalPatterns.html).

### Copy number calling

We estimated copy number profiling over 10-kb windows with Patchwork (http://patchwork.r-forge.r-project.org/)^28^ and calculated the ratio of standardized average depth between normal tissue and tumor tissue (log_2_R ratio). Copy number gains or losses were evaluated with GISTIC 2.0 (https://software.broadinstitute.org/software/cprg/?q=node/31)^29^ using default parameters. The purity and ploidy of each tumor were calculated using ABSOLUTE (http://archive.broadinstitute.org/cancer/cga/absolute)^30^.

### BFB and chromothripsis inference

We inferred BFB events by detecting fold-back inversion and telomere loss. Fold-back inversions^31^ were detected based on three criteria: (1) the single inversions were without reciprocal support-read clusters, (2) the inversion caused a copy number change (*q* < 0.001), and (3) the two ends of the breakpoints had to be separated by <30 kb. We used criteria described by Korbel and Campbell to infer chromothripsis. Two statistical algorithms were applied to infer chromothripsis: (1) Clustering of breakpoints: Let $\left\{ {x_1,x_2, \cdots ,x_n} \right\}$ be the location of breakpoints on a chromosome, ordered from the lowest to the highest. The null model of random breakpoint locations implies that the distances between adjacent breakpoints,$\left\{ {x_2 - x_1,x_3 - x_2, \cdots ,x_n - x_{n - 1}} \right\}$, should be distributed according to an exponential distribution with mean $\mathop {\sum}\nolimits_1^{n - 1} {\left( {x_{i + 1} - x_i} \right)/n - 1}$ which can be readily evaluated using a goodness-of-fit test. (2) Randomness of DNA fragment joins: We counted the numbers of rearrangements that have a deletion-type, tandem duplication-type, head-to-head-inverted, and tail-to-tail-inverted orientation respectively. In a region of chromothripsis, we would expect these four types of SVs to be distributed as a multinomial distribution with equal frequency 0.25. After identifying potential chromothripsis events, we would chose the final real chromothripsis that exhibited a pattern of copy number oscillation between two or three states on chromosomal arms, with >10 rearrangements^32^.

### Inference of kataegis

We inferred kataegis based on four stringent criteria described by Nik-Zainal et al.: (1) the presence of hypermutated genomic regions consisting of a few hundred base pairs and separated by tens of unmutated kilobases, (2) mutation clusters generally colocalized with rearrangement breakpoints, (3) most mutations were derived from the same parental chromosome within the region; and (4) enrichment of C > T and C > G mutations within the region^33^.

### Double minute detection

We used DMFinder (https://github.com/mhayes20/DMFinder) to detect double minute chromosomes in WGS data^34^. Using copy number and SV predictions, this framework incorporated them into a graph-based algorithm capable of finding amplicons that are linked together and that might be double minutes in a tumor genome.

### Identifying SV hotspots

We used an approach described by Glodzik et al. to identify SV hotspots and using the PCF algorithm to determine genomic regions exhibiting rearrangement density much higher than that observed in neighboring genomic regions^11^. SV breakpoints were sorted according to reference-genome coordinates, and an intermutation distance between two genome-sorted breakpoints was calculated and log_10_-transformed, followed by the use of these transformed values in the PCF algorithm. We applied the PCF method to three categories of TDs and deletions separately to explore regions with a rearrangement density exceeding twice the whole-genome background density and involving a minimum of six samples.

### Immunohistochemistry

Immunohistochemistry was performed using a Real EnVision detection system (Dako; Agilent Technologies, Santa Clara, CA, USA). Slides were incubated with a primary antibody to *ZFP36L2* (dilution: 1:50; BS2278, Bioworld Technology, St. Louis Park, MN, USA) and a biotinylated secondary antibody and enzyme conjugate, followed by staining with 3,3′-diaminobenzidine and counterstaining with hematoxylin. The extent of *ZFP36L2* staining was scored by assigning the percentage of positive tumor cells (0, none; 1, <20% of positive-stained cells; 2, 20−50% of positive-stained cells; and 3, >50% of positive-stained cells).

### Cell culture

NCI-N87 cells were purchased from American Type Culture Collection (Manassas, VA, USA), SNU-719 cells were purchased from Cobioer (Nanjing, China). HGC-27 cells were purchased from Cell Bank of Chinese Academy of Sciences (Shanghai, China). GES-1 cells were gotten from Peking University Cancer Hospital (Beijing, China). The mycoplasma contamination of all the cell lines was tested as negative. Cell lines were authenticated by short tandem repeat technology. NCI-N87 and SNU-719 cells were cultured in 1640 medium (Gibco-BRL, Grand Island, NY, USA) with 10% fetal bovine serum (Gibco-BRL, Grand Island, NY, USA), 100 U/ml penicillin and 100 μg/ml streptomycin (Macgene, Beijing, China). HGC-27 and GES-1 cells were cultured in DMEM medium (Gibco-BRL, Grand Island, NY, USA) with 10% fetal bovine serum (Gibco-BRL, Grand Island, NY, USA), 100 U/ml penicillin and 100 μg/ml streptomycin (Macgene, Beijing, China). Cells were maintained at 37 °C/5% CO_2_.

### Polymerase chain reaction (PCR)

2XEasyTag PCR SuperMix (Transgen Biotech, Beijing, China) were used to amplify the DNA. DNA with designed primers was amplified via PCR for 30 cycles, the PCR products were run in 1% agarose and sequenced with Sanger method. The primers were listed in Supplementary Data 7.

### Real-time PCR

Total RNA was extracted from the cell lines using TRIzol (Invitrogen, Carlsbad, CA, USA) and subjected to real-time PCR. cDNA was synthesized using the cDNA reverse transcription kit (Invitrogen, Grand Island, NY, USA), and quantitation of *ZFP36L2* was conducted with an ABI PRISM 7500 System (Applied Biosystems, Foster City, CA, USA). The relative expression level of each gene was normalized to the amount of the same cDNA using the 2-ΔΔCt method and was further compared to its own control. The primer sequence is listed in Supplementary Data 5. Each experiment was repeated three times.

### Transfection and colony formation

*ZFP36L2* overexpression vectors and shRNA against *ZFP36L2* were constructed by GeneChem (Shanghai, China). Vectors were transfected into GC cells using Fugene HD (Promega, Madison, WI, USA), and cells were selected with G418 (Invitrogen, Carlsbad, CA, USA) for 2 weeks. Stably transfected cells were then seeded on 60-mm plates, and the colonies (with >50 cells per colony) were counted after two weeks. Colony formation was analyzed after staining with crystal violet.

### Cell proliferation assay

Cell proliferation was examined in real time using the xCELLigence RTCA DPlus System (ACEA Biosciences, San Diego, CA, USA). The xCELLigence system allows continuous quantitative monitoring of cellular behavior including proliferation by measuring electrical impedance. Cells were seeded at 3000 cells/well into E-Plate 16-well plates. Proliferation was continuously monitored every 15 min over a time period of 96 h. Data analysis was carried out using RTCA Software supplied with the instrument.

### Xenografts assays

Animal experiments were performed in accordance with the National Institutes of Health Guide for the Care and Use of Laboratory Animals with protocols approved by the Animal Care and Use Committee at Peking University Cancer Hospital/Institute. A total of 5×10^6^ NCI-N87 cells coated with Matrigel (BD, San Jose, CA, USA) in 0.1 ml PBS were subcutaneously injected into five nude mice (BALB/c, 4 weeks old, female). Xenograft tumor-bearing mice were sacrificed at 50 days. Tumor xenografts were isolated and weighted.

### Luciferase-reporter assays

We used the pGL3-promoter luciferase-reporter system (Promega, Madison, WI, USA), with the enhancer regions cloned upstream of the pGL3-promoter region. The enhancer-luciferase constructs were then cotransfected along with a control *Renilla* luciferase construct into cells using Fugene HD (Promega, Madison, WI, USA). The luciferase signal was first normalized to the *Renilla* luciferase signal and then to the signal from cells transfected with the empty pGL3 plasmid. Primers used for cloning are listed in Supplementary Data 5.

### The Capture-4C experiment

About 4 million cells were cross-linked with 2% final concentration formaldehyde (Sigma, St Louis, MO, USA) for 10 min at room temperature (RT), then quenched by adding 0.2 M cold glycine, and lysed with cold lysis buffer for 15 min at 4 °C. Cells were centrifuged for 15 min/500 × *g*/4 °C and the supernatant was carefully removed without disturbing the pellet. The pellet nuclei were digested by restriction enzyme *Dpn*II (NEB, Ipswich, MA, USA) for a total of 16 h with incubation on thermomixer. The digests were placed on a 65 °C block for 20 min to heat inactivate the restriction enzyme and then the digest was cooled on ice. Ligation Mix was added to the digest for 22 h at 4 °C, then Proteinase K (Sigma) was added and incubated at 65 °C overnight. Then the DNA purification was carried out by ethanol precipitation. The DNA library was then sonicated using the Biorupter system to break up DNA fragments at 200−500 bp. The sonicated DNA sample was indexed by using NEBnext DNA Library Prep kit (NEB, Ipswich, MA, USA) for end repair, dA labeling, and adaptor ligation. After DNA clean-up steps the adaptor-ligated material (1 μg) was denatured and hybridized with 5 pmol biotinylated capture probe. The captured DNA was purified with streptavidin beads, and the beads were used as templates for generation of capture-C libraries. NEB Q5 Master Mix (NEB, Ipswich, MA, USA) was used to amplify the libraries to generate enough DNA for sequencing. The mixture went through 14 PCR cycles, and PCR products were purified with PCR purification kit (Roche, Basel, Schweiz). The primer used in this experiment is listed in Supplementary Data 8.

### CRISPR/Cas9-mediated deletion of the enhancer region

All sgRNA sequences are listed in Supplementary Data 8. sgRNAs were cloned into pspCas9 (BB)-2A-Puro (Addgene #62988). Vectors were transfected into GC cells using Fugene HD (Promega, Madison, WI, USA), and cells were selected with puromycin (Invitrogen, Carlsbad, CA, USA) for 2 weeks. To detect deletion of the e5 and e11 enhancers, genomic DNA was first extracted and then used for PCR using 2XEasyTag PCR SuperMix (Transgen Biotech) with the primers listed in Supplementary Data 8.

### Quantification and statistical analysis

All statistical tests were performed in R (version 3.4.2). The nonparametric Mann−Whitney *U* test, Fisher’s exact test and Student’s *t* test were used to compare between groups. We also used the log-rank test to perform survival analysis. For all statistical tests used, we assumed that there was independence between data. Box plots show median values and middle quartiles. Using the SPSS 13.0 software (SPSS Inc., Chicago, IL, USA), the significance of immunohistochemistry images was evaluated with the chi-square test.

### Reporting summary

Further information on experimental design is available in the Nature Research Reporting Summary linked to this article.

## Supplementary information

Supplementary Information

Description of Additional Supplementary Files

Supplementary Data 1

Supplementary Data 2

Supplementary Data 3

Supplementary Data 4

Supplementary Data 5

Supplementary Data 6

Supplementary Data 7

Supplementary Data 8

Reporting Summary

## Data Availability

The sequencing data of this paper have been deposited in the European Genome-phenome Archive (accession number: EGAS00001003512, dataset, EGAD00001004811). Data that support the findings of this study are available from HK WGS cohort (http://web.hku.hk/~suetyi/), Catalogue of Somatic Mutations in Cancer (COSMIC) (http://cancer.sanger.ac.uk/cancergenome/projects/cosmic/) and the dataset of topological domains (http://chromosome.sdsc.edu/mouse/hi-c/IMR90.domain.tar.gz).

## References

[1] Cancer Genome Atlas Research N. (2014). Comprehensive molecular characterization of gastric adenocarcinoma. Nature.

[2] Cristescu R (2015). Molecular analysis of gastric cancer identifies subtypes associated with distinct clinical outcomes. Nat. Med..

[3] Wang K (2014). Whole-genome sequencing and comprehensive molecular profiling identify new driver mutations in gastric cancer. Nat. Genet..

[4] Menghi F (2016). The tandem duplicator phenotype as a distinct genomic configuration in cancer. Proc Natl Acad Sci USA.

[5] Nik-Zainal S (2016). Landscape of somatic mutations in 560 breast cancer whole-genome sequences. Nature.

[6] Zhang X (2016). Identification of focally amplified lineage-specific super-enhancers in human epithelial cancers. Nat. Genet..

[7] Falchetti M (2008). Gastric cancer with high-level microsatellite instability: target gene mutations, clinicopathologic features, and long-term survival. Hum. Pathol..

[8] Li X (2016). Distinct subtypes of gastric cancer defined by molecular characterization include novel mutational signatures with prognostic capability. Cancer Res..

[9] Lawrence MS (2013). Mutational heterogeneity in cancer and the search for new cancer-associated genes. Nature.

[10] Menghi F (2018). The tandem duplicator phenotype is a prevalent genome-wide cancer configuration driven by distinct gene mutations. Cancer Cell.

[11] Glodzik D (2017). A somatic-mutational process recurrently duplicates germline susceptibility loci and tissue-specific super-enhancers in breast cancers. Nat. Genet..

[12] Yonemori K (2017). ZFP36L2 promotes cancer cell aggressiveness and is regulated by antitumor microRNA-375 in pancreatic ductal adenocarcinoma. Cancer Sci..

[13] Lin DC (2018). Identification of distinct mutational patterns and new driver genes in oesophageal squamous cell carcinomas and adenocarcinomas. Gut.

[14] Hnisz D (2013). Super-enhancers in the control of cell identity and disease. Cell.

[15] Cancer Genome Atlas Research N. (2017). Integrated genomic characterization of oesophageal carcinoma. Nature.

[16] Kagey MH (2010). Mediator and cohesin connect gene expression and chromatin architecture. Nature.

[17] Dixon JR (2012). Topological domains in mammalian genomes identified by analysis of chromatin interactions. Nature.

[18] Secrier M (2017). Corrigendum: Mutational signatures in esophageal adenocarcinoma define etiologically distinct subgroups with therapeutic relevance. Nat. Genet..

[19] Alexandrov LB (2013). Signatures of mutational processes in human cancer. Nature.

[20] Baca SC (2013). Punctuated evolution of prostate cancer genomes. Cell.

[21] Stephens PJ (2011). Massive genomic rearrangement acquired in a single catastrophic event during cancer development. Cell.

[22] McKenna A (2010). The Genome Analysis Toolkit: a MapReduce framework for analyzing next-generation DNA sequencing data. Genome Res..

[23] Yang L (2013). Diverse mechanisms of somatic structural variations in human cancer genomes. Cell.

[24] Cibulskis K (2013). Sensitive detection of somatic point mutations in impure and heterogeneous cancer samples. Nat. Biotechnol..

[25] Wala JA (2018). SvABA: genome-wide detection of structural variants and indels by local assembly. Genome Res..

[26] Ramos AH (2015). Oncotator: cancer variant annotation tool. Hum. Mutat..

[27] Alexandrov LB, Nik-Zainal S, Wedge DC, Campbell PJ, Stratton MR (2013). Deciphering signatures of mutational processes operative in human cancer. Cell Rep..

[28] Mayrhofer M, DiLorenzo S, Isaksson A (2013). Patchwork: allele-specific copy number analysis of whole-genome sequenced tumor tissue. Genome Biol..

[29] Mermel CH (2011). GISTIC2.0 facilitates sensitive and confident localization of the targets of focal somatic copy-number alteration in human cancers. Genome Biol..

[30] Carter SL (2012). Absolute quantification of somatic DNA alterations in human cancer. Nat. Biotechnol..

[31] Campbell PJ (2010). The patterns and dynamics of genomic instability in metastatic pancreatic cancer. Nature.

[32] Korbel JO, Campbell PJ (2013). Criteria for inference of chromothripsis in cancer genomes. Cell.

[33] Nik-Zainal S (2012). Mutational processes molding the genomes of 21 breast cancers. Cell.

[34] Hayes M, Li J (2015). An integrative framework for the identification of double minute chromosomes using next generation sequencing data. BMC Genet..

