# Supplementary material for: Whole-genome sequencing reveals novel tandem-duplication hotspots and a prognostic mutational signature in gastric cancer
Source: Nat Commun. 2019 May 2;10:2037. doi: 10.1038/s41467-019-09644-6 (PMC6497673; doi:10.1038/s41467-019-09644-6)
Supplement: Supplementary file 1 — Supplementary Information [file 41467_2019_9644_MOESM1_ESM.pdf]

Supplemental information

Whole-genome sequencing reveals novel tandem-duplication hotspots and a prognostic mutational signature in gastric cancer

Rui Xing et al.

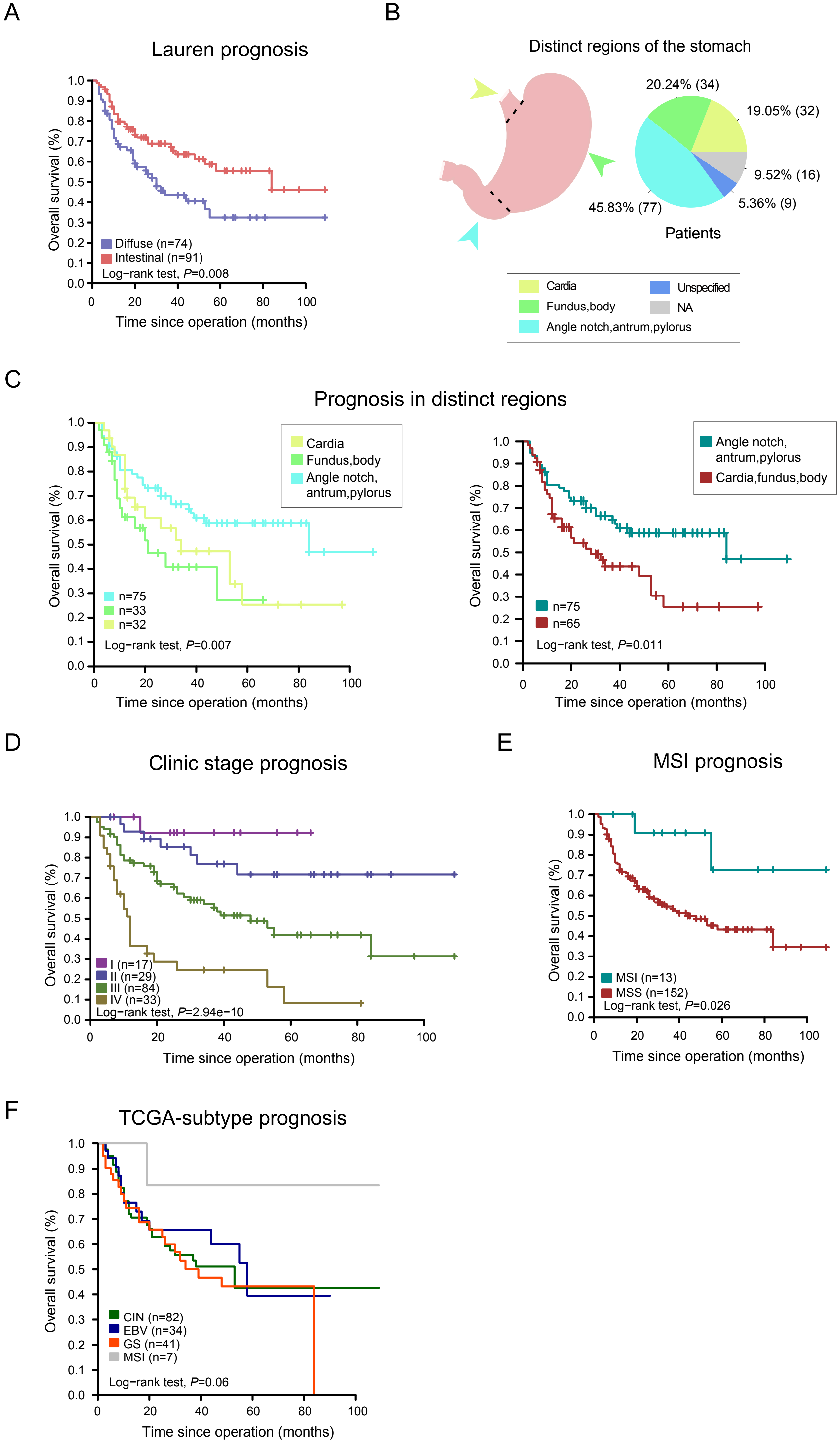
A
Lauren prognosis
Overall survival (%)
Time since operation (months)
Diffuse (n=74)
Intestinal (n=91)
Log−rank test, P=0.008
B
Distinct regions of the stomach
20.24% (34)
19.05% (32)
9.52% (16)
5.36% (9)
45.83% (77)
Patients
Cardia
Fundus,body
Angle notch,antrum,pylorus
Unspecified
NA
C
Prognosis in distinct regions
Cardia
Fundus,body
Angle notch, antrum,pylorus
n=75
n=33
n=32
Log−rank test, P=0.007
Angle notch, antrum,pylorus
Cardia,fundus,body
n=75
n=65
Log−rank test, P=0.011
D
Clinic stage prognosis
I (n=17)
II (n=29)
III (n=84)
IV (n=33)
Log−rank test, P=2.94e−10
E
MSI prognosis
MSI (n=13)
MSS (n=152)
Log−rank test, P=0.026
F
TCGA-subtype prognosis
CIN (n=82)
EBV (n=34)
GS (n=41)
MSI (n=7)
Log−rank test, P=0.06

Supplementary Figure 1. Kaplan-Meier survival curves display survival outcomes of (A) Lauren classification, (C) distinct regions, (D) clinical stage, (E) MSI status and (F) TCGA-subtype of the stomach. (B) Specimens from 168 GC patients were categorized into five groups along with their localization.

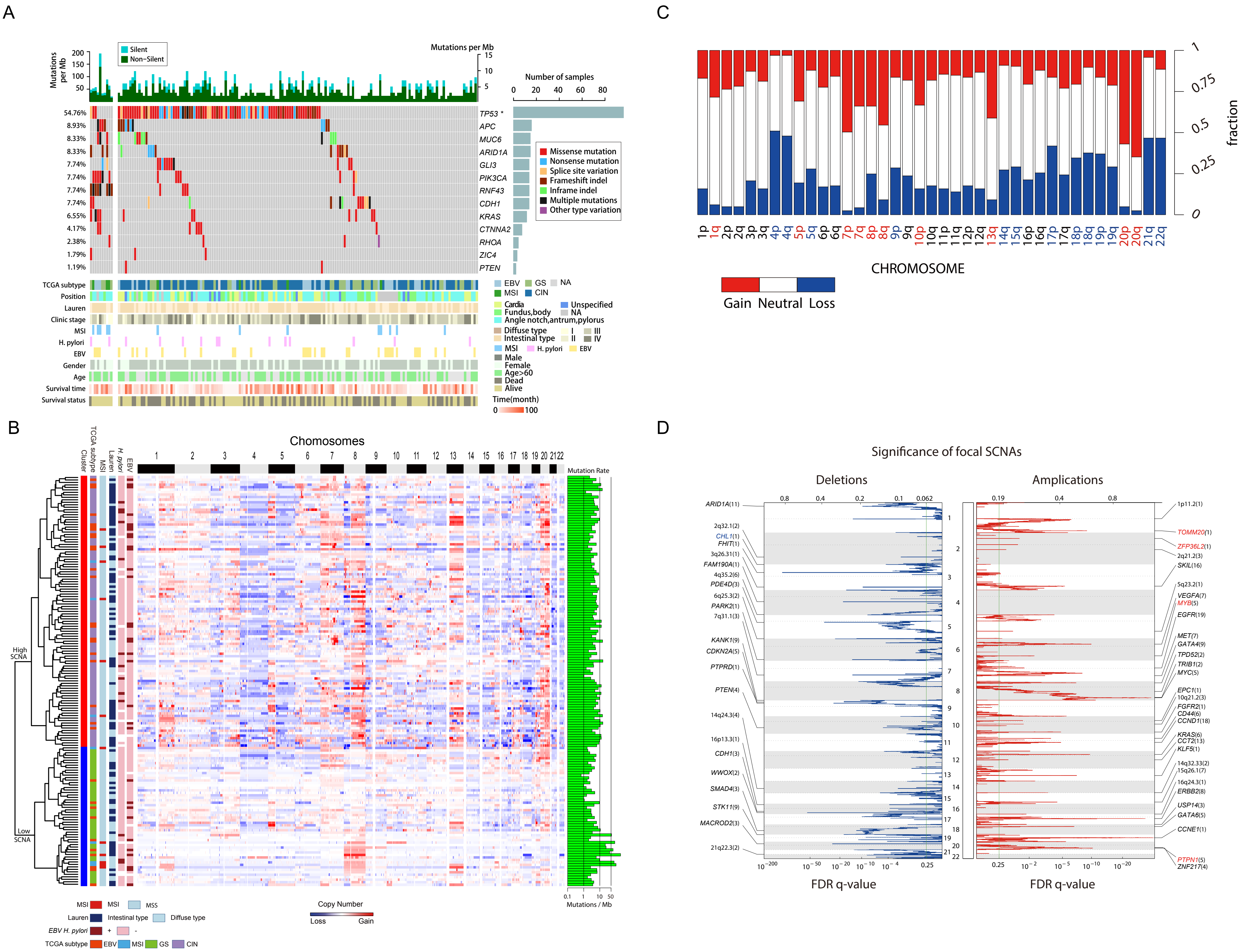
A
Mutations per Mb
Silent
Non-Silent
Number of samples
TP53 *
APC
MUC6
ARID1A
GLI3
PIK3CA
RNF43
CDH1
KRAS
CTNNA2
RHOA
ZIC4
PTEN
54.76%
8.93%
8.33%
8.33%
7.74%
7.74%
7.74%
7.74%
6.55%
4.17%
2.38%
1.79%
1.19%
Missense mutation
Nonsense mutation
Splice site variation
Frameshift indel
Inframe indel
Multiple mutations
Other type variation
TCGA subtype
Position
Lauren
Clinic stage
MSI
H. pylori
EBV
Gender
Age
Survival time
Survival status
EBV
GS
NA
MSI
CIN
Cardia
Unspecified
Fundus,body
NA
Angle notch,antrum,pylorus
Diffuse type
Intestinal type
I
II
III
IV
MSI
H. pylori
EBV
Male
Female
Age>60
Dead
Alive
Time(month)
0
100
B
Chomosomes
Cluster
TCGA subtype
MSI
Lauren
H. pylori
EBV
Mutation Rate
High SCNA
Low SCNA
Mutations / Mb
Copy Number
Loss
Gain
MSI
MSI
MSS
Lauren
Intestinal type
Diffuse type
EBV H. pylori
+
-
TCGA subtype
EBV
MSI
GS
CIN
C
fraction
CHROMOSOME
Gain
Neutral
Loss
D
Significance of focal SCNAs
Deletions
Amplifications
FDR q-value
FDR q-value
ARID1A(11)
2q32.1(2)
CHL1(1)
FHIT(1)
3q26.31(1)
FAM190A(1)
4q35.2(6)
PDE4D(3)
6q25.3(2)
PARK2(1)
7q31.1(3)
KANK1(9)
CDKN2A(5)
PTPRD(1)
PTEN(4)
14q24.3(4)
16p13.3(1)
CDH1(3)
WWOX(2)
SMAD4(3)
STK11(9)
MACROD2(3)
21q22.3(2)
1p11.2(1)
TOMM20(1)
ZFP36L2(1)
2q21.2(3)
SKIL(16)
5q23.2(1)
VEGFA(7)
MYB(5)
EGFR(19)
MET(7)
GATA4(9)
TPD52(2)
TRIB1(2)
MYC(5)
EPC1(1)
10q21.2(3)
FGFR2(1)
CD44(6)
CCND1(18)
KRAS(6)
CCT2(13)
KLF5(1)
14q32.33(2)
15q26.1(7)
16q24.3(1)
ERBB2(8)
USP14(3)
GATA6(5)
CCNE1(1)
PTPN1(5)
ZNF217(4)

Supplementary Figure 2. Landscape of mutation and CNV in GC. (A) Significantly mutated genes and driver mutations in 168 gastric cancer cases. Somatic mutations shown across the 13 gastric cancer genes (one significantly mutated genes TP53 and 12 driver genes) for 168 GC samples (10 hypermutated cases on the left and 158 non-hypermutated cases are displayed on the right). Multiple mutations refer to one gene harbored two or more protein-coding mutations in one sample. (B) In the heatmap, SCNAs in tumors (vertical axis) are plotted by chromosomal location (horizontal axis). For copy number based clustering, tumours are clustered based on threshold of copy number at reoccurring alteration common peaks (24 focal amplification regions, 23 focal deletion regions) from 165 cases and TCGA cohort. Clustering is done in R based on Euclidean distance using Ward clustering with ward.D2 algorithms. (C) Arm-level copy number analysis. The bar graphs show the frequency of arm level copy number alterations in molecular subtypes. Red refers to copy number gain and blue. (D) Identification of focal recurrent copy number gains (red) and losses (blue) by the GISTIC 2.0 method. Annotated peaks have an FDR < 0.25 and encompass 18 or fewer genes. Peaks are annotated with candidate driver oncogenes, tumor suppressors. The number of genes within each peak is shown next to driver genes or cytobands.

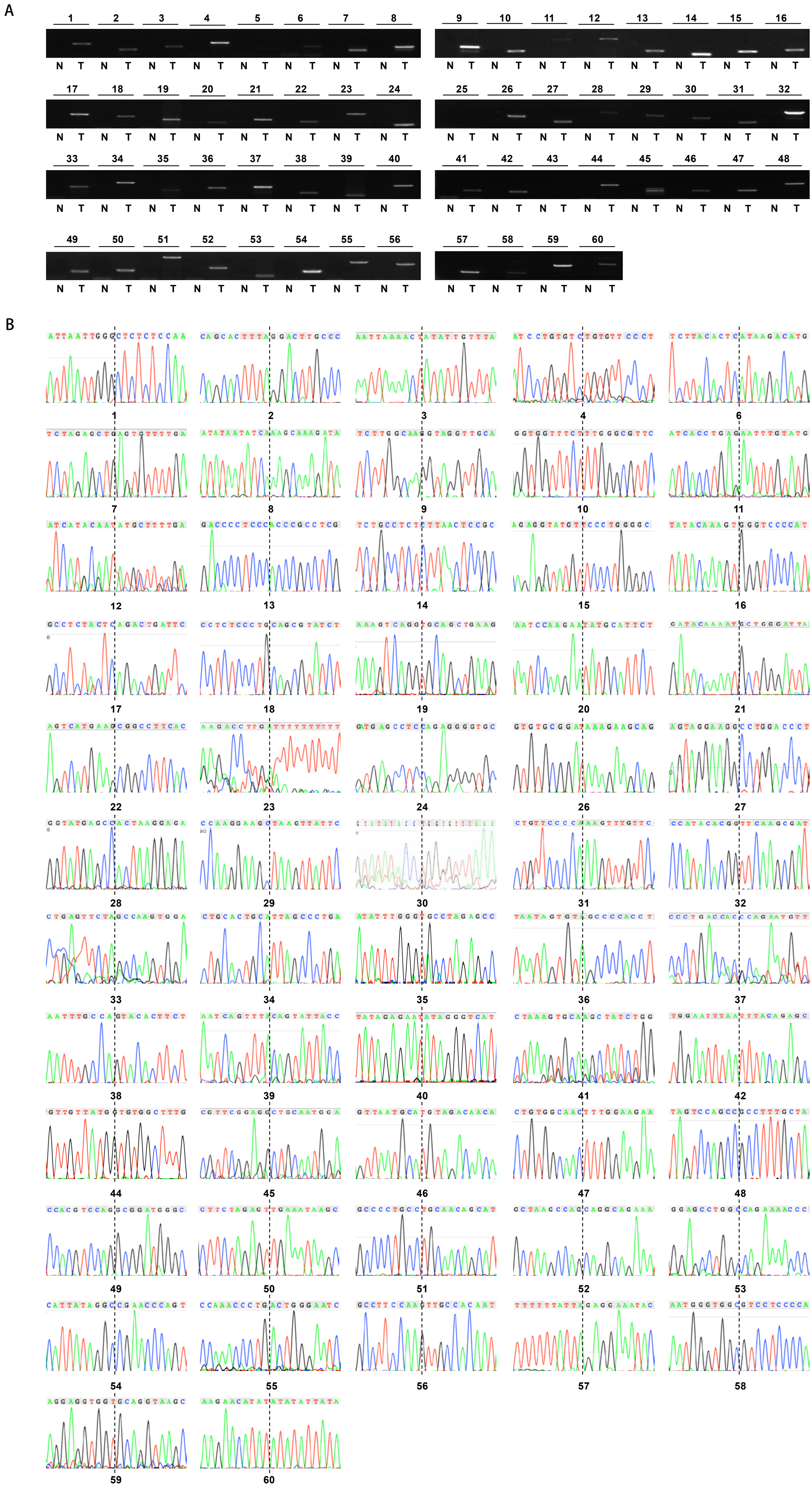
A
B

Supplementary Figure 3. Validation of SVs by PCR and Sanger sequence. (A) Gel pictures of PCR amplification of genomic DNA using primers list in Supplementary Data 7. (B) Sanger sequence of PCR amplification of genomic DNA using primers list in Supplementary Data 7.

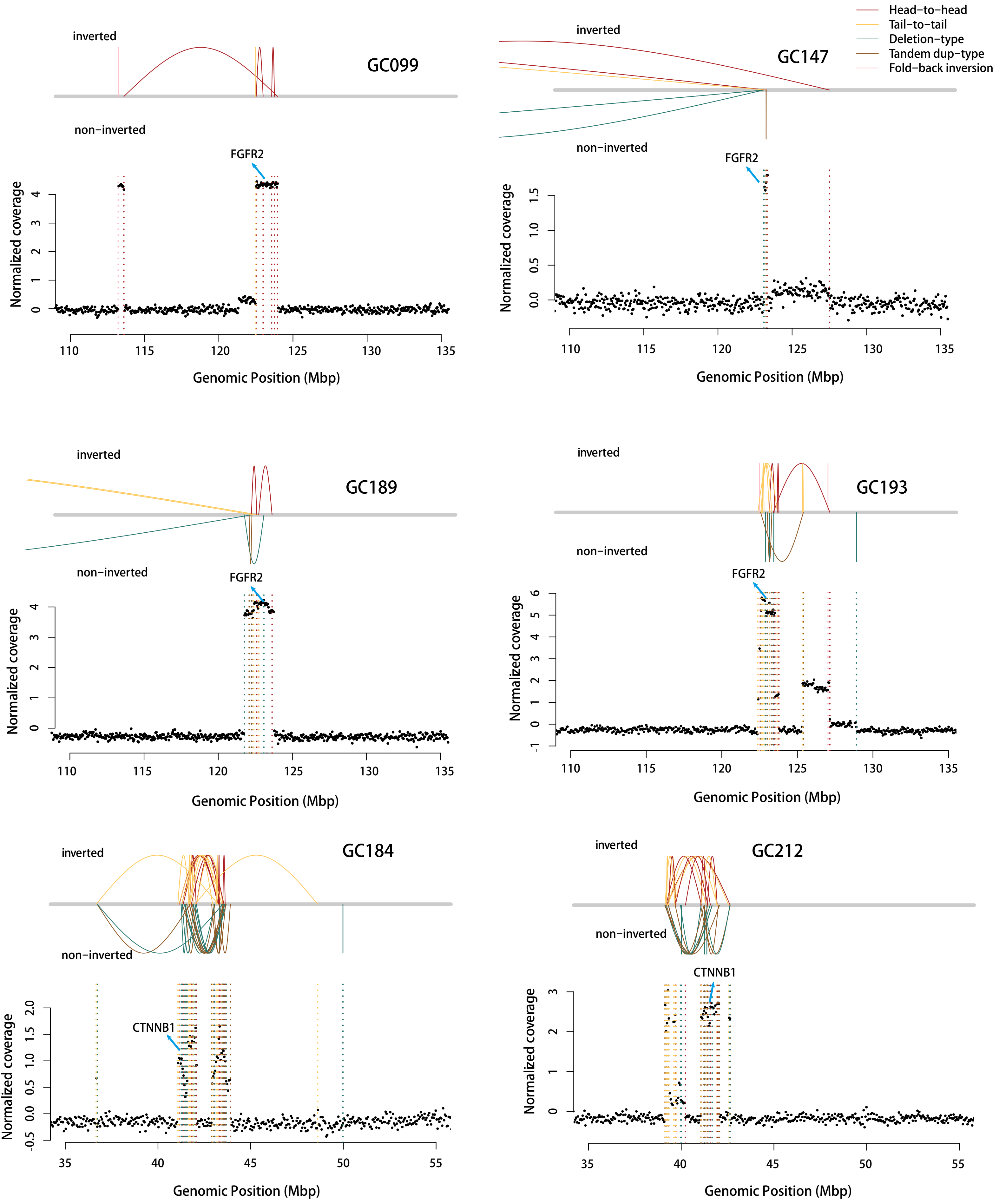

GC099
GC147
GC189
GC193
GC184
GC212
inverted
non−inverted
Head−to−head
Tail−to−tail
Deletion−type
Tandem dup−type
Fold−back inversion
FGFR2
CTNNB1
Normalized coverage
Genomic Position (Mbp)

Supplementary Figure 4. Double minute patterns with focal amplifications of *FGFR2* and *CTNNB1*. The upper panel shows intra-chromosome rearrangements, and the bottom panel shows the normalized coverage around *FGFR2* and *CTNNB1*.

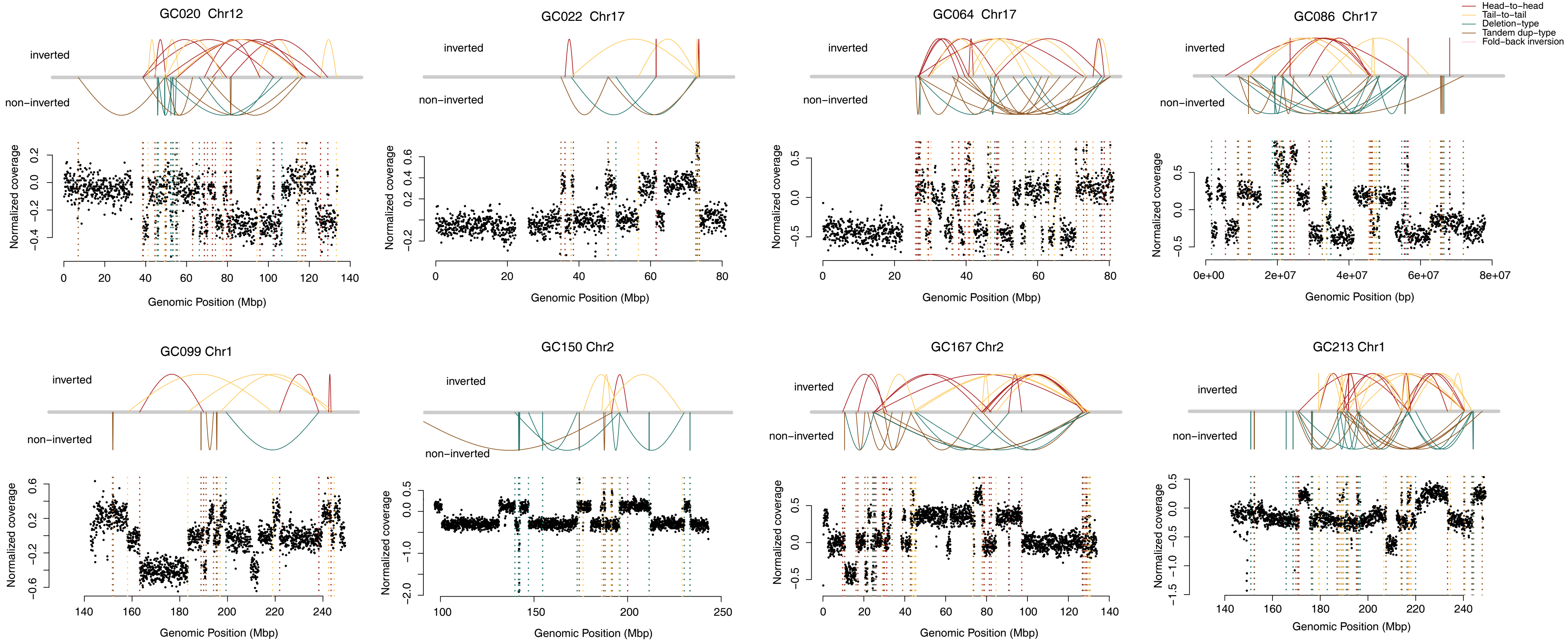

GC020 Chr12
GC022 Chr17
GC064 Chr17
GC086 Chr17
GC099 Chr1
GC150 Chr2
GC167 Chr2
GC213 Chr1
inverted
non−inverted
Normalized coverage
Genomic Position (Mbp)
Genomic Position (bp)
Head−to−head
Tail−to−tail
Deletion−type
Tandem dup−type
Fold−back inversion

Supplementary Figure 5. Eight GC specimens with chromothripsis. The upper panel shows diverse types of intra-chromosome rearrangements, and the bottom panel shows normalized coverage for each marker.

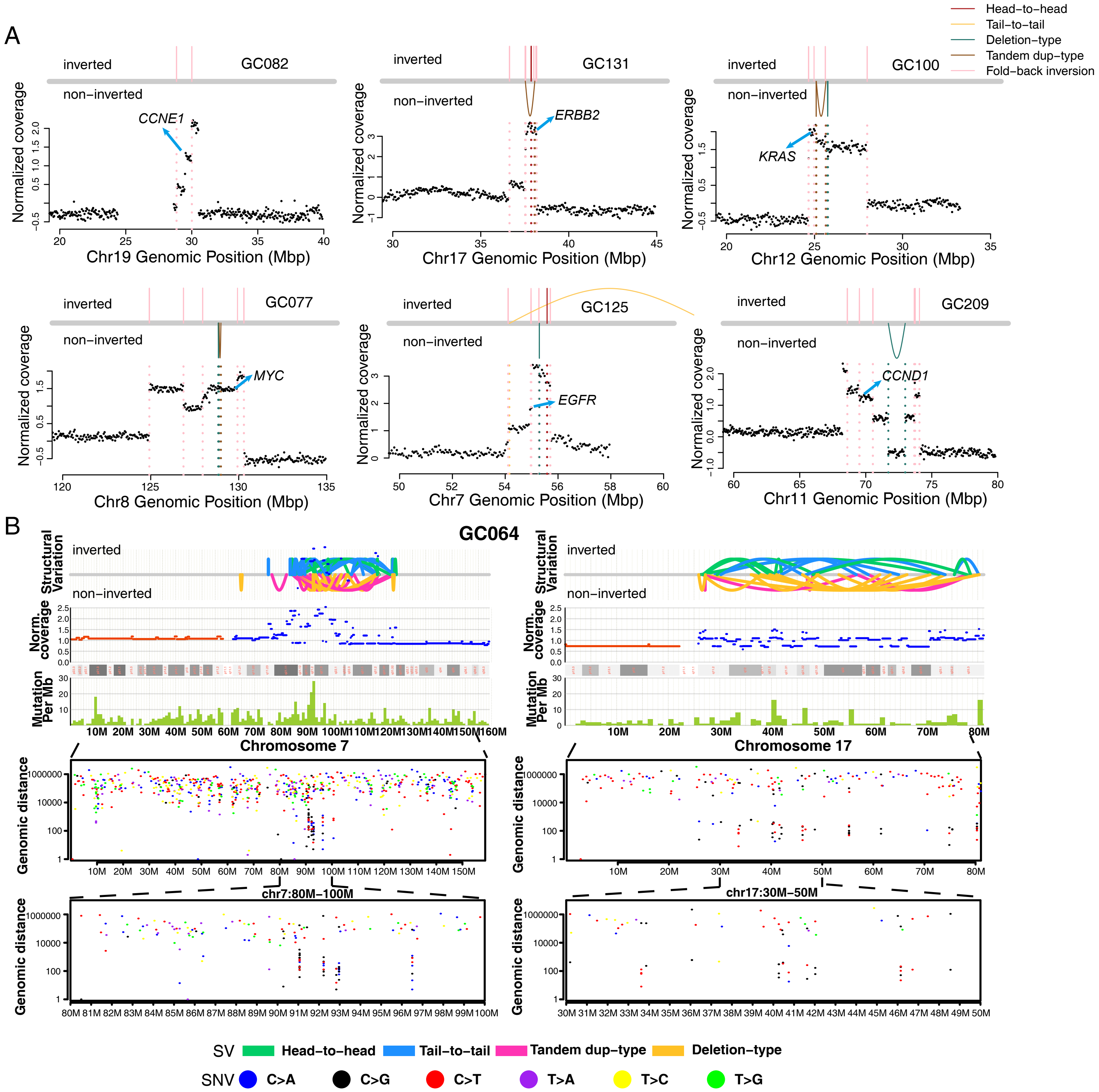

A
Head−to−head
Tail−to−tail
Deletion−type
Tandem dup−type
Fold−back inversion
inverted
non−inverted
Normalized coverage
GC082
CCNE1
Chr19 Genomic Position (Mbp)
GC131
ERBB2
Chr17 Genomic Position (Mbp)
GC100
KRAS
Chr12 Genomic Position (Mbp)
GC077
MYC
Chr8 Genomic Position (Mbp)
GC125
EGFR
Chr7 Genomic Position (Mbp)
GC209
CCND1
Chr11 Genomic Position (Mbp)
B
GC064
Structural Variation
Norm. coverage
Mutation Per Mb
Chromosome 7
Chromosome 17
Genomic distance
chr7:80M−100M
chr17:30M−50M
SV Head−to−head Tail−to−tail Tandem dup−type Deletion−type
SNV C>A C>G C>T T>A T>C T>G

Supplementary Figure 6. BFB events and kataegis across 168 GC specimens. (A) Representative maps show BFB events resulting in the amplification of oncogenes, such as *CCNE1*, *CCND1*, *EGFR, MYC, ERBB2,* and *KRAS*. **(B)** Kataegis on chromosomes 7 and 17 in GC064. The upper panel shows different SV types, and the bottom panel shows distributions of different types of point mutations.

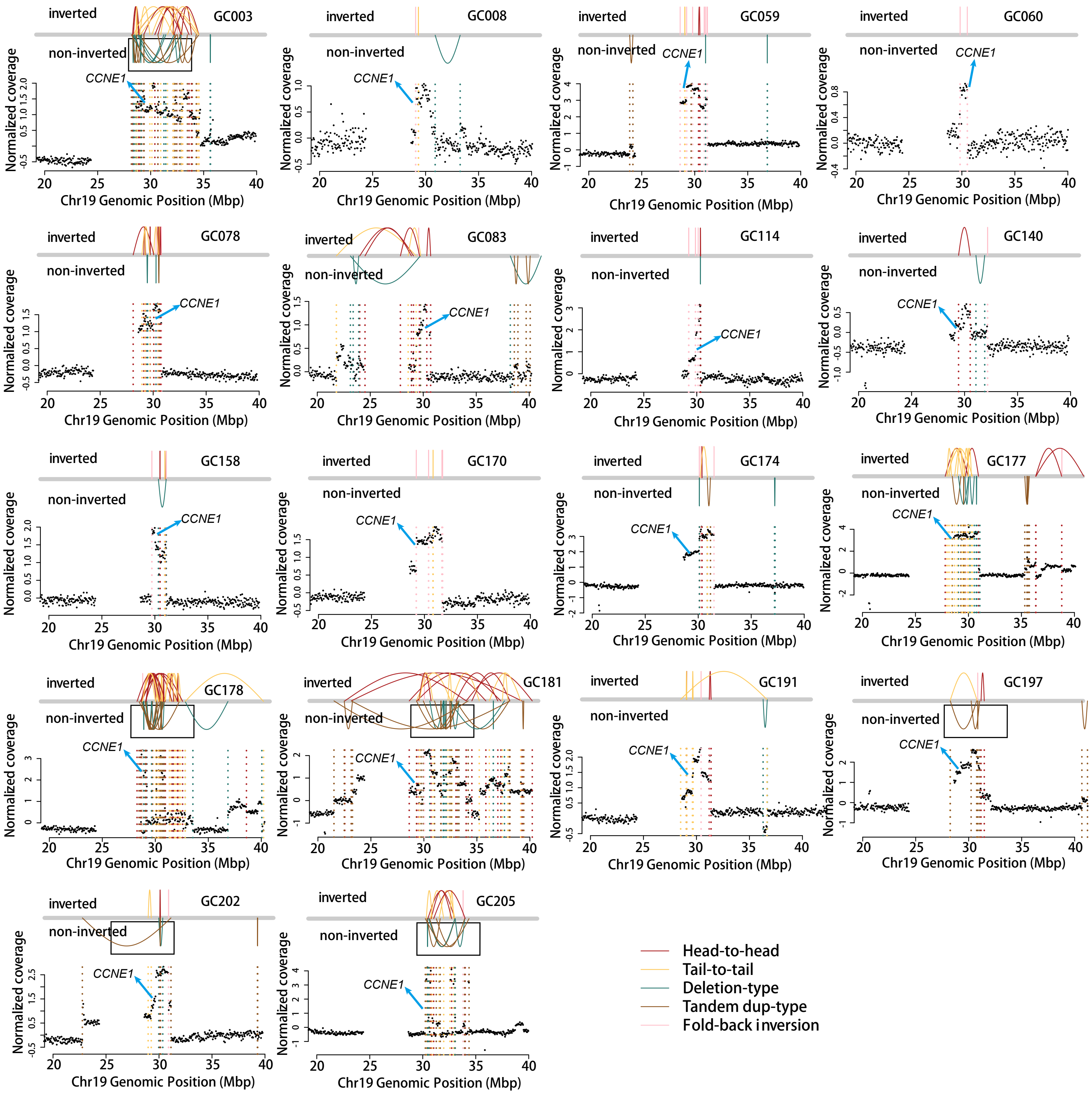

GC003
GC008
GC059
GC060
GC078
GC083
GC114
GC140
GC158
GC170
GC174
GC177
GC178
GC181
GC191
GC197
GC202
GC205
inverted
non-inverted
CCNE1
Normalized coverage
Chr19 Genomic Position (Mbp)
Head-to-head
Tail-to-tail
Deletion-type
Tandem dup-type
Fold-back inversion

Supplementary Figure 7. Amplification of *CCNE1* by BFB event in 18 GC specimens. The upper panel shows diverse types of intra-gene rearrangements in chromosome 19, and the bottom panel shows normalized coverage for each marker.

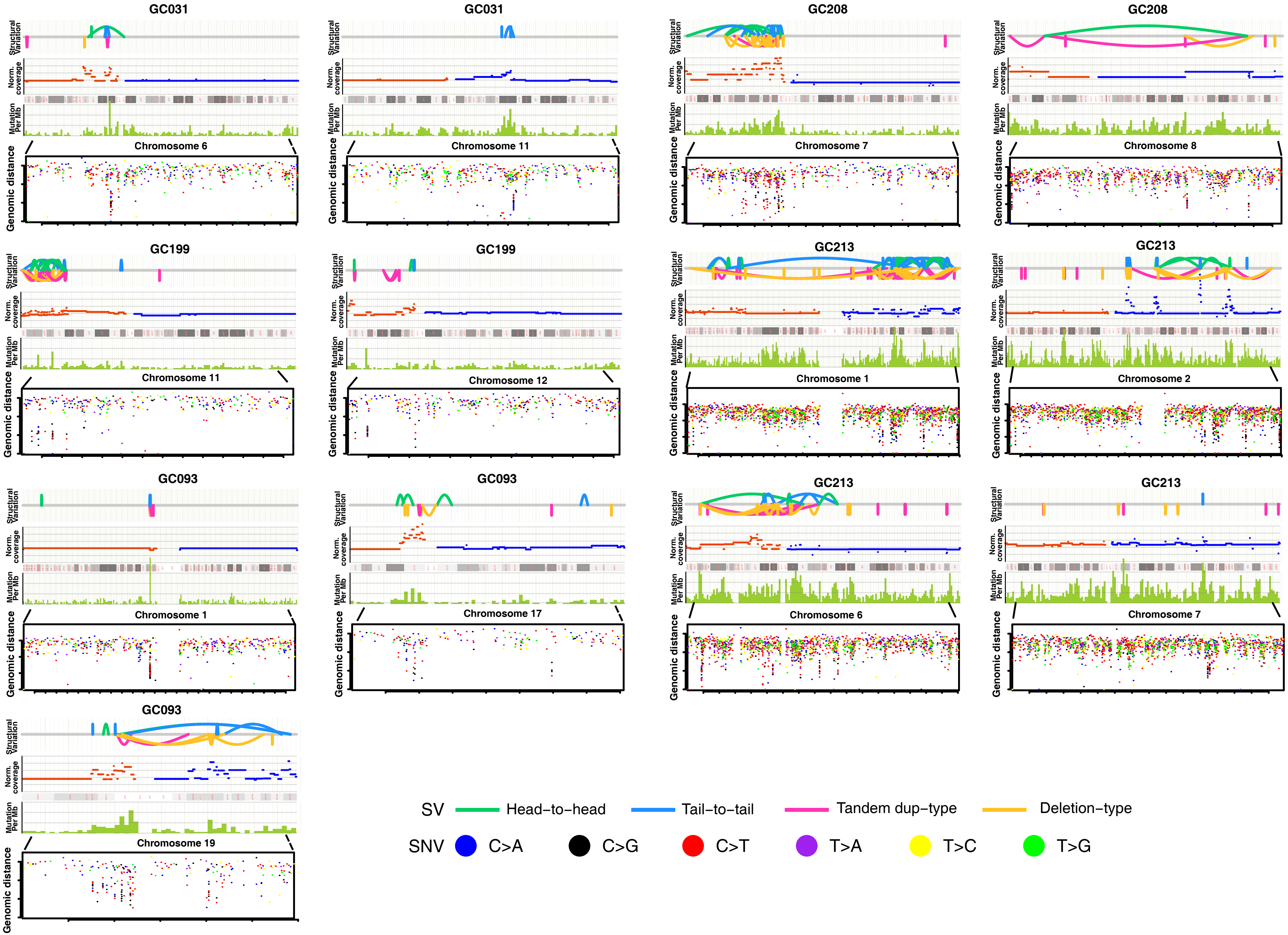
GC031
Chromosome 6
GC031
Chromosome 11
GC208
Chromosome 7
GC208
Chromosome 8
GC199
Chromosome 11
GC199
Chromosome 12
GC213
Chromosome 1
GC213
Chromosome 2
GC093
Chromosome 1
GC093
Chromosome 17
GC213
Chromosome 6
GC213
Chromosome 7
GC093
Chromosome 19
Structural Variation
Norm. coverage
Mutation Per Mb
Genomic distance
SV
Head–to–head
Tail–to–tail
Tandem dup–type
Deletion–type
SNV
C>A
C>G
C>T
T>A
T>C
T>G

Supplementary Figure 8. Kataegis in five GC specimens. The upper panel shows different SV types, and the bottom panel shows distributions of different types of point mutations.

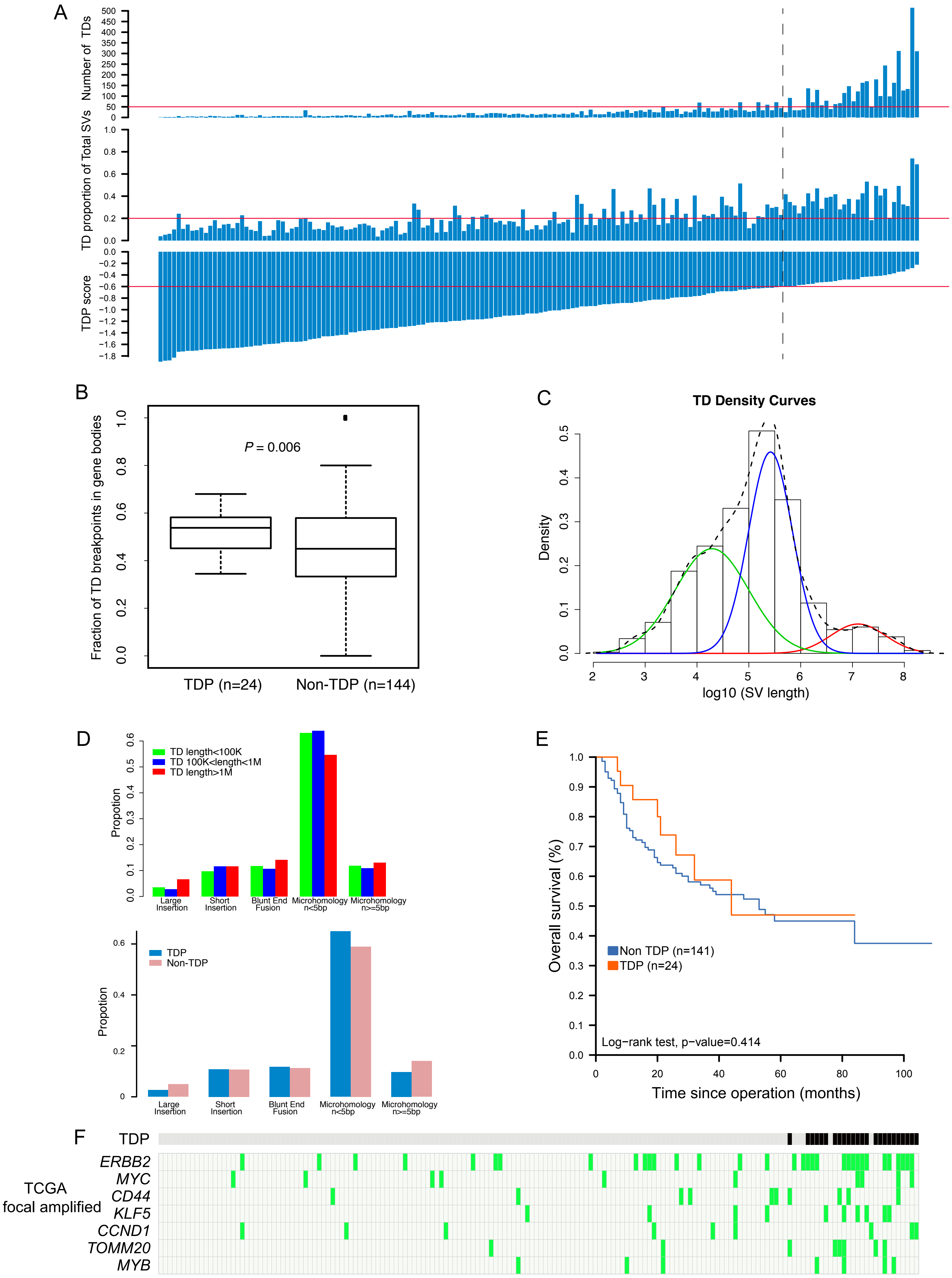

A
Number of TDs
TD proportion of Total SVs
TDP score
B
Fraction of TD breakpoints in gene bodies
P = 0.006
TDP (n=24)
Non-TDP (n=144)
C
TD Density Curves
Density
log10 (SV length)
D
TD length<100K
TD 100K<length<1M
TD length>1M
Propotion
Large Insertion
Short Insertion
Blunt End Fusion
Microhomology n<5bp
Microhomology n>=5bp
TDP
Non-TDP
E
Overall survival (%)
Non TDP (n=141)
TDP (n=24)
Log−rank test, p−value=0.414
Time since operation (months)
F
TDP
TCGA focal amplified
ERBB2
MYC
CD44
KLF5
CCND1
TOMM20
MYB

Supplementary Figure 9. Genomic features of TDs in GC. (A) Barplots show three qualification used to define TDP in GC. (B) TDs are more likely to engage gene bodies than intergenic regions in TDP GCs. Center line represents the median of fraction of TD breakpoints and *P*-value was derived from Wilcoxon rank-sum test. (C) Size distributions for TDs, which generally exhibit three peaks at ~10 kb, 250 kb, and 1 Mb. (D) Sequence analysis of TD-breakpoint junctions across TDP and non-TDP GCs and three size categories. (E) Kaplan-Meier survival curves display no survival outcomes associated with TDP. (F) Summary of TD hotspots occurring within TCGA focal-amplified peaks.

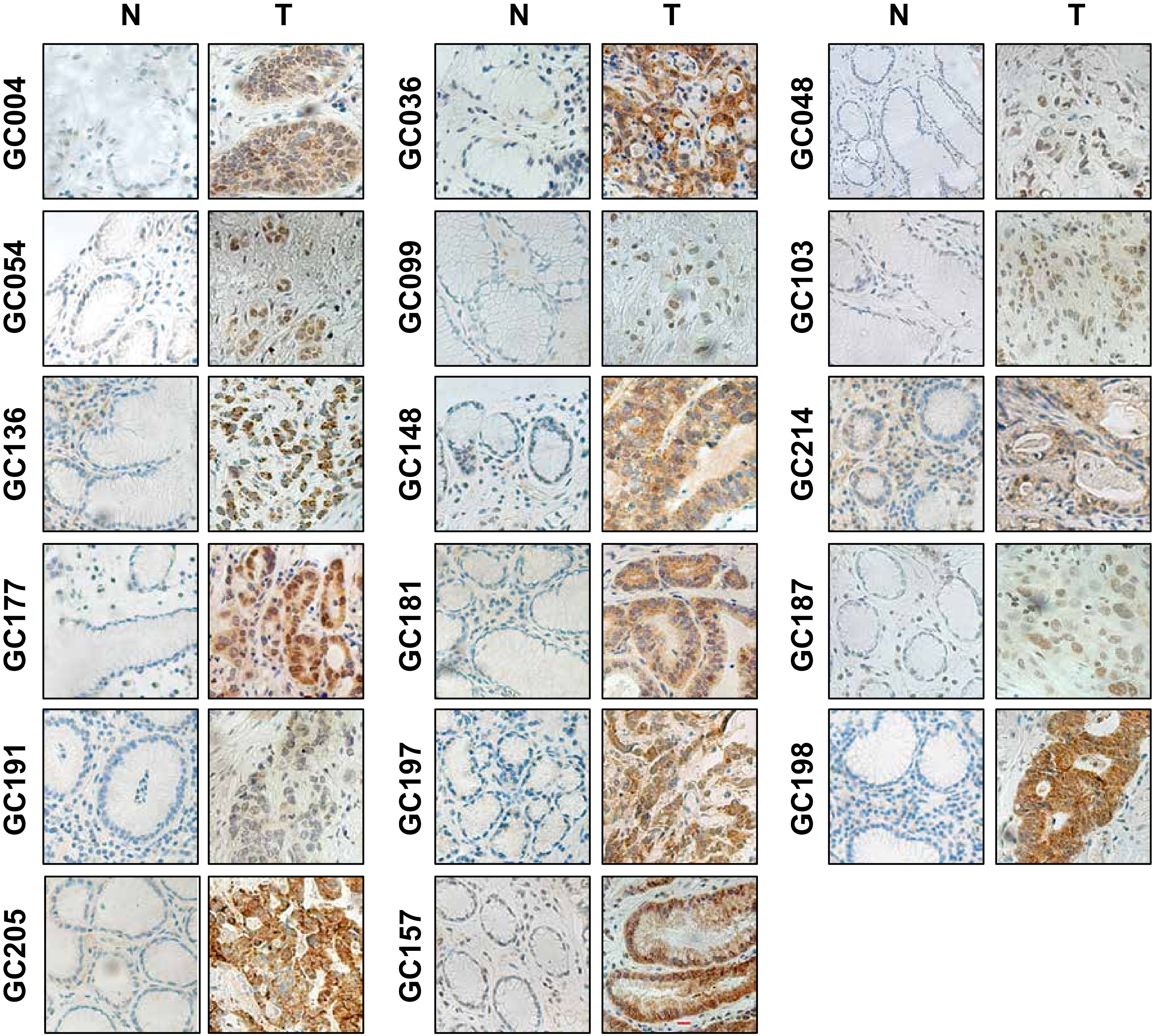
N
T
N
T
N
T
GC004
GC036
GC048
GC054
GC099
GC103
GC136
GC148
GC214
GC177
GC181
GC187
GC191
GC197
GC198
GC205
GC157

Supplementary Figure 10. Immunohistochemistry detection of *ZFP36L2* expression in 17 GC samples with amplification. Scale bars 25μM.

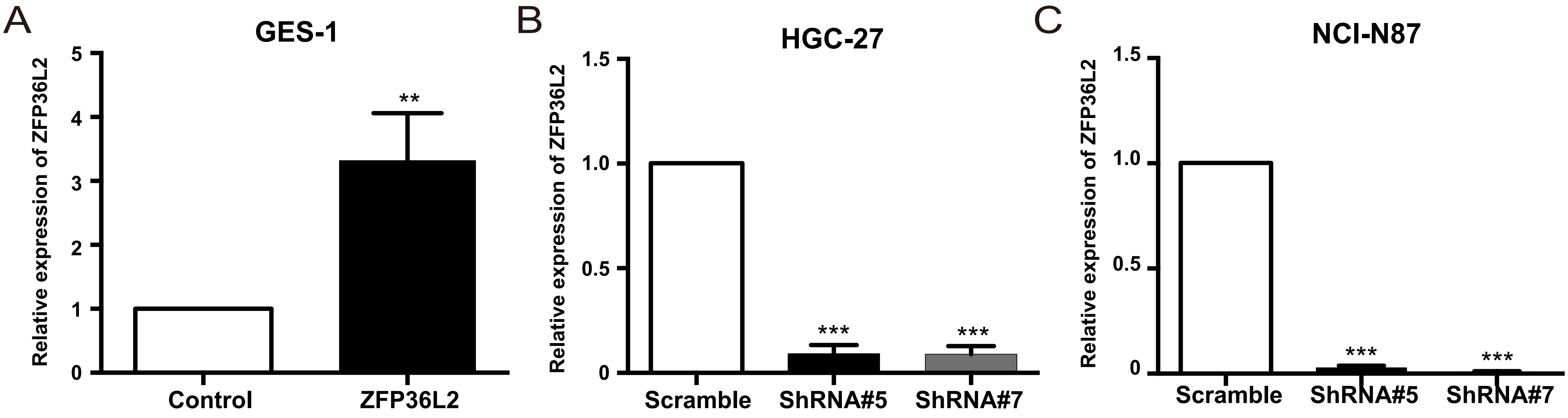

A
GES-1
Relative expression of ZFP36L2
5
4
3
2
1
0
**
Control
ZFP36L2
B
HGC-27
Relative expression of ZFP36L2
1.5
1.0
0.5
0
***
***
Scramble
ShRNA#5
ShRNA#7
C
NCI-N87
Relative expression of ZFP36L2
1.5
1.0
0.5
0
***
***
Scramble
ShRNA#5
ShRNA#7

Supplementary Figure 11. Realtime-PCR detected the effect of *ZFP36L2* overexpressed in GES-1 cells (A), **knocked down in HGC-27 cells** (B) **and NCI-N87 cells** (C). Error bars represent ± s.d. of three experiments (*P*-values were derived from t tests, $^{**}P \leqslant 0.01$; $^{***}P \leqslant 0.001$).

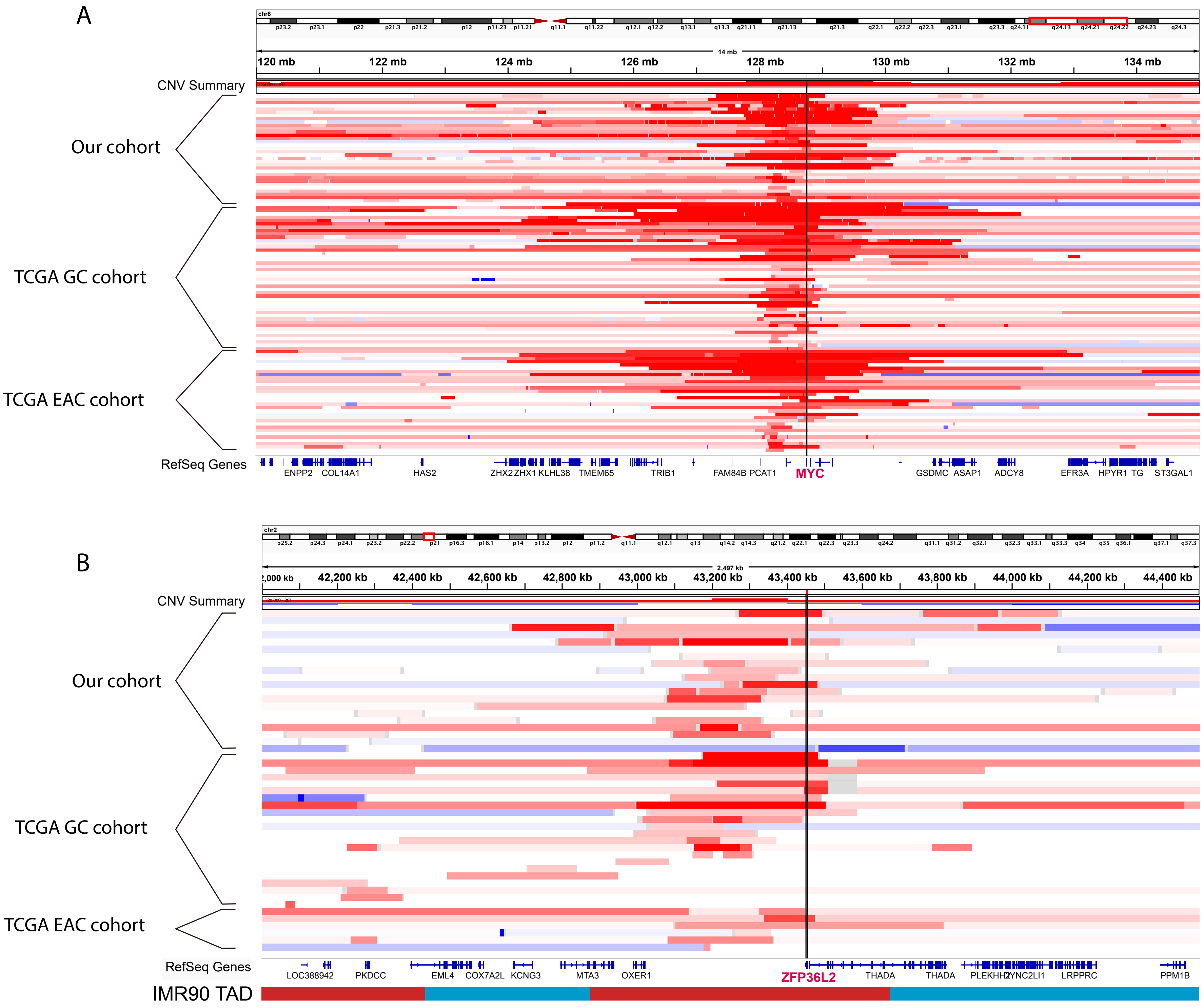
A
chr8
120 mb
122 mb
124 mb
126 mb
128 mb
130 mb
132 mb
134 mb
CNV Summary
Our cohort
TCGA GC cohort
TCGA EAC cohort
RefSeq Genes
ENPP2
COL14A1
HAS2
ZHX2
ZHX1
KLHL38
TMEM65
TRIB1
FAM84B
PCAT1
MYC
GSDMC
ASAP1
ADCY8
EFR3A
HPYR1
TG
ST3GAL1
B
chr2
42,200 kb
42,400 kb
42,600 kb
42,800 kb
43,000 kb
43,200 kb
43,400 kb
43,600 kb
43,800 kb
44,000 kb
44,200 kb
44,400 kb
CNV Summary
Our cohort
TCGA GC cohort
TCGA EAC cohort
RefSeq Genes
LOC388942
PKDCC
EML4
COX7A2L
KCNG3
MTA3
OXER1
ZFP36L2
THADA
THADA
PLEKHH2
DYNC2LI1
LRPPRC
PPM1B
IMR90 TAD

Supplementary Figure 12. Plots showing focal amplifications around gene *ZFP36L2* and *MYC* in our cohort, the TCGA GC cohort, and the TCGA EAC cohort. (A) Focally amplified region (black line) around *MYC* viewed by IGV is plotted along the chromosome. (B) Focally amplified region (black line) around *ZFP36L2* viewed by IGV is plotted along the chromosome. The topologically associated domains (TAD) are showed as adjacent red and blue bars in the bottom panel.

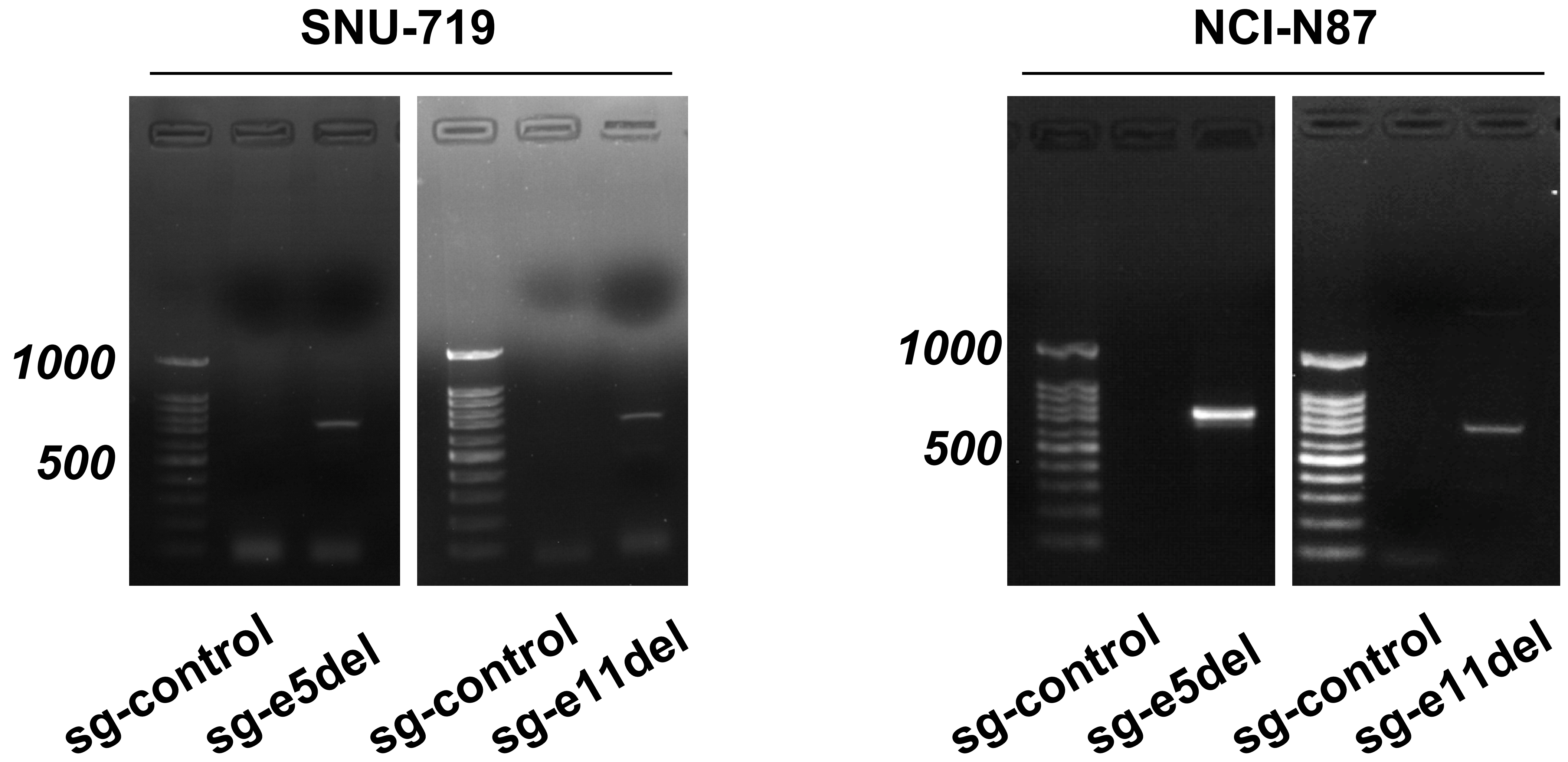
SNU-719
NCI-N87
1000
500
1000
500
sg-control
sg-e5del
sg-control
sg-e11del
sg-control
sg-e5del
sg-control
sg-e11del

Supplementary Figure 13. Full gel pictures of PCR amplification of genomic DNA using primers outside the e5 and e11 enhancer region in (left) SNU-719 and (right) NCI-N87 cells with CRISPR/Cas9 mediated deletion of the e5 and e11 enhancer. sg-Control: empty plasmid; sg-e5del and sg-e11del: pairs of sgRNAs recognizing boundaries of the e5 and e11 enhancer region.

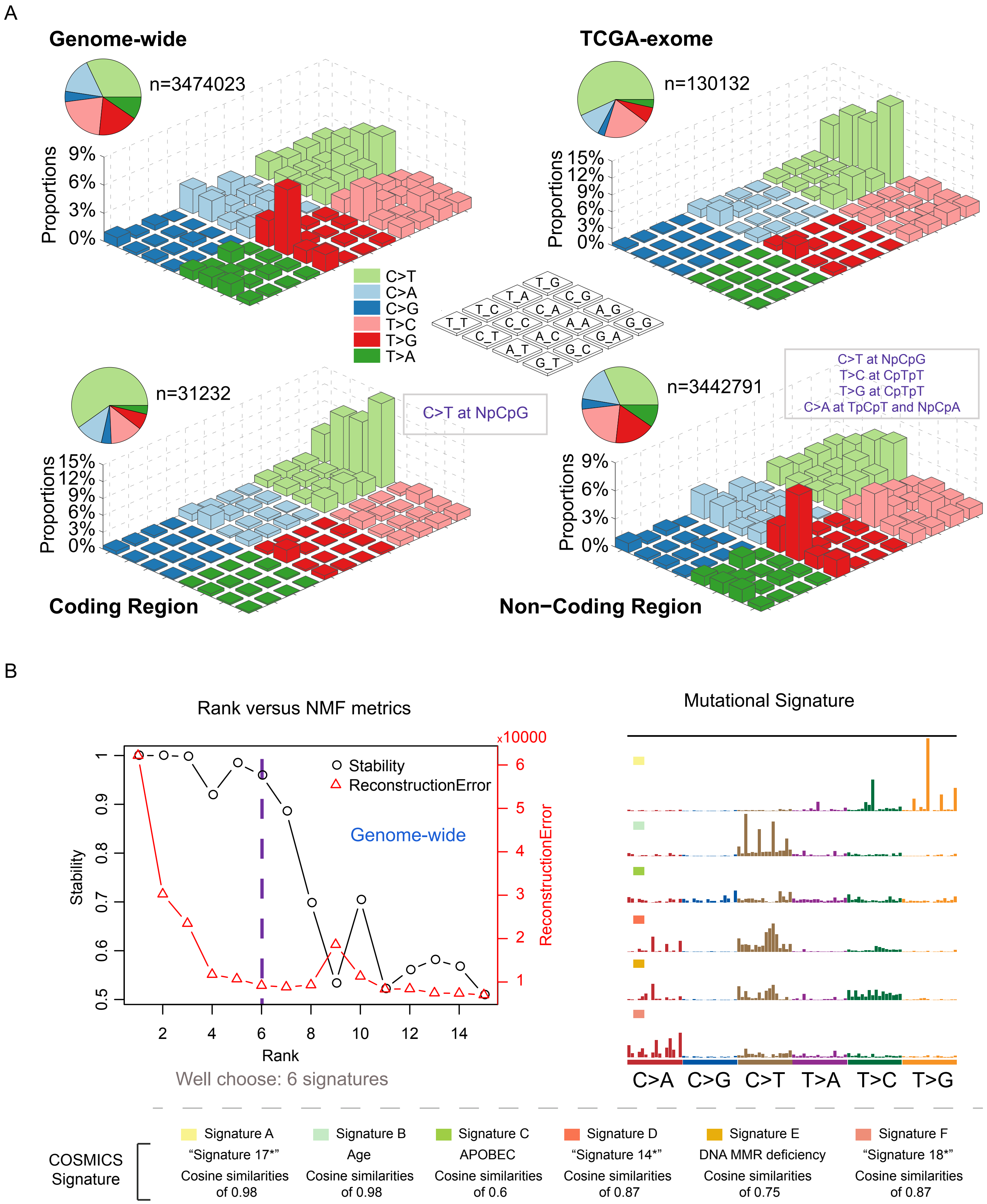

A
Genome-wide
n=3474023
Proportions
9%
6%
3%
0%
TCGA-exome
n=130132
15%
12%
9%
6%
3%
0%
C>T
C>A
C>G
T>C
T>G
T>A
T_G
T_A
C_G
T_C
C_A
A_G
T_T
C_C
A_A
G_G
C_T
A_C
G_A
A_T
G_C
G_T
n=31232
C>T at NpCpG
n=3442791
C>T at NpCpG
T>C at CpTpT
T>G at CpTpT
C>A at TpCpT and NpCpA
Coding Region
Non−Coding Region
B
Rank versus NMF metrics
x10000
Stability
ReconstructionError
Genome-wide
Rank
Well choose: 6 signatures
Mutational Signature
C>A
C>G
C>T
T>A
T>C
T>G
COSMICS Signature
Signature A
"Signature 17*"
Cosine similarities of 0.98
Signature B
Age
Cosine similarities of 0.98
Signature C
APOBEC
Cosine similarities of 0.6
Signature D
"Signature 14*"
Cosine similarities of 0.87
Signature E
DNA MMR deficiency
Cosine similarities of 0.75
Signature F
"Signature 18*"
Cosine similarities of 0.87

Supplementary Figure 14. Mutational signatures in GC. (A) Mutation-spectrum analysis of genome-wide (our cohort), coding-region (our cohort), noncoding region (our cohort), and TCGA-exome sequences. Base substitutions were divided into six subtypes to represent the six possible base changes (each subtype is represented by a different color). The proportion was calculated as the number of somatic SNVs divided by the number of somatic SNVs that contained the specified trinucleotide. Different trinucleotide types are highlighted in lilac. (B) Stability plot shows six signatures present in 168 GC data. Signatures are displayed according to the 96-substitution classification, with the X-axis representing mutation types, and the Y-axis representing the trinucleotide frequency in each mutation type. Similarity between COSMIC signatures and those derived from this study are shown in the bottom panel

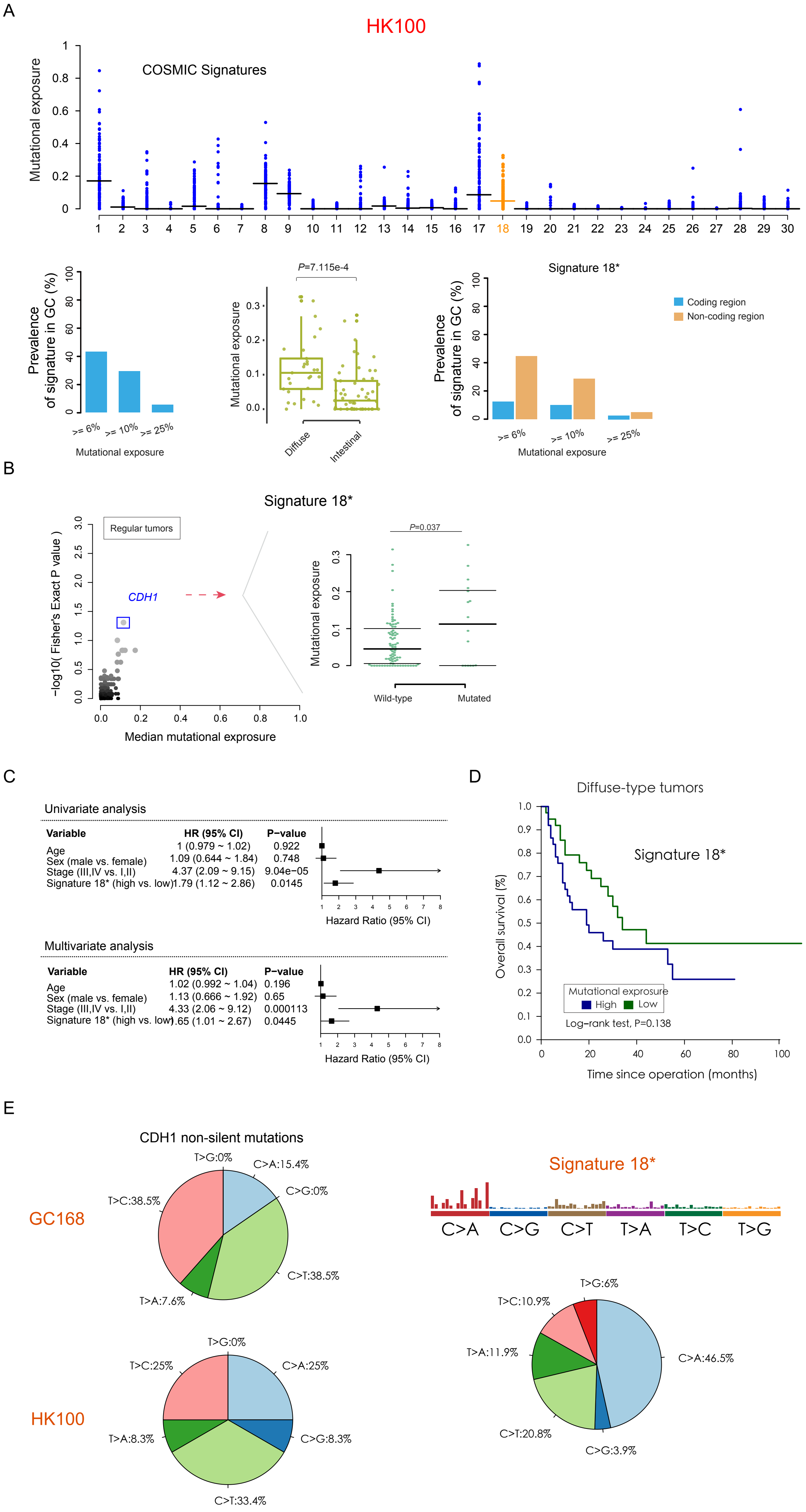
A
HK100
COSMIC Signatures
Mutational exposure
Prevalence of signature in GC (%)
Mutational exposure
P=7.115e-4
Diffuse
Intestinal
Signature 18*
Coding region
Non-coding region
>= 6%
>= 10%
>= 25%
B
Signature 18*
Regular tumors
CDH1
−log10(Fisher's Exact P value)
Median mutational exprosure
P=0.037
Wild-type
Mutated
C
Univariate analysis
Variable
HR (95% CI)
P−value
Age
1 (0.979 ~ 1.02)
0.922
Sex (male vs. female)
1.09 (0.644 ~ 1.84)
0.748
Stage (III,IV vs. I,II)
4.37 (2.09 ~ 9.15)
9.04e−05
Signature 18* (high vs. low)
1.79 (1.12 ~ 2.86)
0.0145
Hazard Ratio (95% CI)
Multivariate analysis
Variable
HR (95% CI)
P−value
Age
1.02 (0.992 ~ 1.04)
0.196
Sex (male vs. female)
1.13 (0.666 ~ 1.92)
0.65
Stage (III,IV vs. I,II)
4.33 (2.06 ~ 9.12)
0.000113
Signature 18* (high vs. low)
1.65 (1.01 ~ 2.67)
0.0445
Hazard Ratio (95% CI)
D
Diffuse-type tumors
Signature 18*
Overall survival (%)
Mutational exposure
High
Low
Log-rank test, P=0.138
Time since operation (months)
E
CDH1 non-silent mutations
GC168
T>G:0%
C>A:15.4%
C>G:0%
T>C:38.5%
C>T:38.5%
T>A:7.6%
HK100
T>G:0%
T>C:25%
C>A:25%
T>A:8.3%
C>G:8.3%
C>T:33.4%
Signature 18*
C>A
C>G
C>T
T>A
T>C
T>G
T>G:6%
T>C:10.9%
T>A:11.9%
C>A:46.5%
C>T:20.8%
C>G:3.9%

Supplementary Figure 15. Summary of the presence of mutational signature 18* in HK WGS cohort. (A) Mutational contribution of each of 30 COSMIC signatures across GCs. In the bottom panel, presence of signature 18* with different contributions was revealed (*left*). Relationship between signature 18* and diffuse-type GC was shown (*middle*). One dot represents one sample. More, prevalence of signatures 18* across the HK cohort and between noncoding and coding regions (*right*). (B) Mutational-exposure analysis revealed an association between somatic *CDH1* mutations and mutational signature 18* in regular GCs. Genes mutated in >4% of samples were chosen from HK100. *P*-values were derived from Fisher's exact tests. The contribution of signature 18* was compared in wild-type versus mutated tumors. Center line of boxplot represents the median of mutational exposure of signature 18*. *P*-values were derived from *t* tests. Genes with a p-value (p) < 0.05 are marked in blue. *CDH1* was the only gene showing a significant difference. (C) Univariate and multivariate Cox regression analyses for age, sex, TNM staging and signature 18*. HR, 95% CI, and *P*-value was displayed. (D) Kaplan-Meier survival curves show the survival outcomes of signature 18* in diffuse-type GC. High mutational-signature contribution represents mutational-signature contributions >=25%. (E) Distribution of CDH1 non-silent mutations and signature 18* displayed using the six substitution subtypes: C>A, C>G, C>T, T>A, T>C, and T>G.
