# Supplementary material for: Whole-genome sequencing reveals novel tandem-duplication hotspots and a prognostic mutational signature in gastric cancer
Source: Nat Commun. 2019 May 2;10:2037. doi: 10.1038/s41467-019-09644-6 (PMC6497673; doi:10.1038/s41467-019-09644-6)
Supplement: Supplementary file 2 — Description of Additional Supplementary Files [file 41467_2019_9644_MOESM2_ESM.pdf]

Description of Additional Supplementary Information

File Name: Supplementary Data 1.
Description: Clinico-pathological data of 168 gastric cancer patients in the discovery cohort.

File Name: Supplementary Data 2.
Description: List of all somatic SV events across 168 GCs.

File Name: Supplementary Data 3.
Description: A) List of in_frame fusions identified in 168 GC genomes. B) List of out_frame fusions in 168 GC genomes.

File Name: Supplementary Data 4.
Description: A) Summary of hotspots identified for TDs. B) Summary of hotspots identified for DELs.

File Name: Supplementary Data 5.
Description: Primers used in the study.

File Name: Supplementary Data 6.
Description: Sequencing depths and coverage.

File Name: Supplementary Data 7.
Description: Primers used for SV validation. All primers are designed by Primer3 using selected 60 SVs identified in GC177 as input. The type of SV and the genes formed SV were also listed.

File Name: Supplementary Data 8.
Description: Primers used for plasmid construction, Realtime-PCR and Cas9.
